# Metabolic and Non-metabolic Roles of Pyruvate Kinase M2 Isoform in Diabetic Retinopathy

**DOI:** 10.1038/s41598-020-64487-2

**Published:** 2020-05-04

**Authors:** Ammaji Rajala, Krutik Soni, Raju V. S. Rajala

**Affiliations:** 1 Department of Ophthalmology, University of Oklahoma Health Sciences Center, Oklahoma, 73104 USA; 2 Department of Physiology, University of Oklahoma Health Sciences Center, Oklahoma, 73104 USA; 3 Department of Cell Biology, University of Oklahoma Health Sciences Center, Oklahoma, 73104 USA; 4 Dean McGee Eye Institute, Oklahoma City, USA

**Keywords:** Cell biology, Neuroscience

## Abstract

The main therapeutic goal for diabetic retinopathy (DR) is to prevent vision loss in patients with diabetes mellitus. Identifying the visual complications at a preclinical juncture will offer an early therapeutic window for diagnosis and intervention. Very recently, we found that pyruvate kinase M2 isoform (PKM2) regulates visual function through regulation of a key enzyme, phosphodiesterase 6β (Pde6β), involved in modulating photoreceptor functions. A recent study showed that the activation of PKM2 protects mitochondrial integrity in diabetic nephropathy. In the present study, we examined the role of PKM2 in DR in a mouse model that has both phenotypes of obesity and type II diabetes. In DR, we found decreased expression of PKM2 and Pde6β expression, but not PKM1. Consistent with decreased Pde6β expression, the *db/db* mice had reduced rod photoreceptor function. We found increased pyruvate kinase activity and a decreased ratio of reduced/oxidized redox in *db/db* mouse retina compared with control retinas. There was no significant difference in the levels of lactate between *db/db* and control mouse retina. Our findings suggest that reduced expression of PKM2 with unchanged PKM1 expression might be responsible for higher pyruvate kinase activity in *db/db* mouse retina. Our studies suggest that PKM2 has a role in DR. The results support that PKM2 may serve as a therapeutic target in the treatment of DR.

## Introduction

Diabetic retinopathy (DR) is one of the principal causes of vision loss that stems from diabetes mellitus (DM)^1,2^. DR is always associated with progressive vision loss and is accompanied by hemorrhages, neovascularization, and macular edema^3^. By the time the vascular abrasions are identified, the majority of DR patients experience severe and irreversible vision loss. It was reported that diabetic patients with angiographically normal retinas experience visual disturbances, including color discrimination and changes in contrast sensitivity^3^. The main therapeutic goal for DR is to prevent vision loss in DM patients. Identifying the visual complications at a preclinical juncture will offer an early therapeutic window for diagnosis and intervention. Several studies with diabetic animal models revealed decreased visual function^4,5^. The molecular mechanism behind this decrease is poorly understood. Both rod (night vision) and cone (daylight vision) photoreceptors are highly metabolic, and their energy demand is comparable to that of rapidly growing tumor cells^6–8^.

Almost 100 years ago, German scientist Otto Warburg discovered that the retina consumes the highest amount of oxygen, equivalent to that of aggressively proliferating tumor cells^6,7^. Differentiated cells utilize glucose for oxidative phosphorylation, whereas proliferating tumor cells take up increased glucose and use it for anabolic processes instead of mitochondrial oxidation in the presence of oxygen; this effect is called the Warburg effect^6^. Investigators have proposed that regulation of pyruvate kinase M2 (PKM2) isoform activity increases the flux through the pentose phosphate pathway through the accumulation of glycolytic intermediates. Glycolysis is indispensable for every cell, and pyruvate kinase enzymatically converts phosphoenolpyruvate (PEP) to pyruvate, which is the last step in glycolysis.

In the retina, we previously reported the expression of the PKM2 isoform^9^, which regulates both metabolic and non-metabolic functions^10^. In tumor cells, replacing the PKM2 isoform with the PKM1 isoform reverses cancer phenotype in an animal model^11^. Very recently, we found that rod photoreceptor cells lacking PKM2 have no effect on retinal morphology, but exhibit a significant loss of rod function at 5 months of age^12^. However, we found cell death in rods lacking PKM2^12^. Very recently, it was reported that PKM2 activation protects mitochondrial integrity in diabetic nephropathy^13^. These studies led to the hypothesis that reduced vision loss in diabetes could be due to decreased levels of PKM2 in diabetes. Thus, we studied the role of PKM2 in DR in a mouse model that has both phenotypes of obesity and type II diabetes.

## Materials and Methods

### Materials

#### Antibodies

Polyclonal PKM1, PKM2, phospho-PDH (S293), and PDH antibodies were acquired from Cell Signaling (Danvers, MA). A monoclonal actin antibody was purchased from Affinity BioReagents (Golden, CO). Goat polyclonal Pde6β antibody and goat secondary antibodies were obtained from Santa Cruz Biotechnology (Santa Cruz, CA). Dr. James F. McGinnis (University of Oklahoma Health Sciences Center) provided the monoclonal 1D4 rhodopsin antibody and Dr. Jian-Xing Ma (University of Oklahoma Health Sciences Center) provided the polyclonal Rpe65 antibody. GAPDH antibody was purchased from ABclonal (Woburn, MA).

#### Animals

Animal experiments were conducted as per the guidelines of the *Association for Research in Vision and Ophthalmology Statement for the Use of Animals in Ophthalmic and Vision Research* and the *NIH Guide for the Care and Use of Laboratory Animals*. The protocols were approved by the IACUC at the University of Oklahoma Health Sciences Center. Breeding pairs of leptin receptor-deficient *db/db* (BKS.Cg-Dock7m+/+ Leprdb/J) mice and age-matched, non–diabetic control (C57BLKS/J) mice were purchased from The Jackson Laboratory (Bar Harbor, Maine). Animal breeding was carried out in the DMEI vivarium. All animals were raised under dim cyclic light (40–60 lux, 12 h dark/light cycle). Diabetes was induced by a series of two injections. At 8 and 9 weeks, C57BL6/J mice were weighed and given intraperitoneal injections (100 mg/kg) of streptozotocin (STZ) in freshly dissolved citrate buffer (10 mmol, pH 4.5). Control animals were given intraperitoneal injections of citrate buffer. Six weeks after STZ administration, mice were used for experiments. Mice with blood glucose levels greater than 250 mg/dL (TrueTrack Smart System; AR-MED Ltd., Egham, UK) were considered hyperglycemic. Ten week-old *db/db* mice were used for experiments. The *db/db* mice with blood sugar greater than 250 mg/dL were confirmed as diabetic mice. Retinas were immediately removed after euthanasia and were frozen in liquid nitrogen. Eye tissues were harvested for biochemistry or immunohistochemistry.

#### Determination of pyridine nucleotides in retinal tissues by cycling assay

The pyridine nucleotides, nicotinamide adenine dinucleotide (NAD+), reduced nicotinamide adenine dinucleotide (NADH), nicotinamide adenine dinucleotide phosphate (NADP+), and reduced nicotinamide adenine dinucleotide phosphate (NADPH), were measured according to the assay described earlier^14^. To extract NAD+ and NADP+, the retina was homogenized in 5 volumes of 0.23 M KH_2_PO_4_ at 100 °C for 1 min, then chilled and neutralized with 5 volumes of 0.2 M KOH. The reaction was centrifuged at 4 °C for 30 min at 20,000 × g. The extracts were used immediately after centrifugation. To extract NADH and NADPH, the retina was homogenized in 0.2 M KOH for 1 min at 100 °C, then immediately neutralized with 0.23 M KH_2_PO_4._ The reaction was centrifuged at 4 °C for 30 min at 20,000 × g. The extracts were used immediately after centrifugation. The extracts were diluted with water to measure oxidized coenzymes, whereas 0.01 M sodium phosphate buffer, pH 7.4 was used to dilute extracts for the measurement of reduced pyridine nucleotides. For assays of NAD+ and NADH, the reaction mixture contained 0.12 M Bicine, pH 7.8, 0.63 M ethanol, 0.058 M niacinamide, 0.197 mM Thiazolyl Blue (MTT), 1.6 mM phenazine ethosulfate (PES), and 0.25 mg alcohol dehydrogenase. For the assay of NADP+ and NADPH, the reaction mixture contained 0.12 M Bicine, pH 7.8, 2.5 mM glucose 6-phosphate, 0.045 M niacinamide, 0.197 mM Thiazolyl Blue, 0.9 mM PES, and 5.0 units of glucose 6-phosphate dehydrogenase. The formation of formazan by the reduction of MTT was measured in a spectrophotometer at 570 nm. The dehydrogenase and the substrate promote the oxidized coenzyme to cycle back to the reduced form. The progressive increase in absorbance at 570 nm is directly relational to the amount of the coenzyme in the assay mixture.

#### Determination of lactate in the retina samples

We measured lactate using the lactate oxidase method (Trinity Biotech, Jamestown, NY). The reaction was carried out between 25–37 °C. The retina was lysed in phosphate buffered-saline (PBS) and subjected to centrifugation to remove the insoluble material. Ten microliters of the sample were added to a 96-well microtiter plate. Then, 200 µl of lactate reagent were added. The plate was incubated for 5–10 min at room temperature. Then, the absorbance was measured at 540 nm. We calculated the concentration of lactate in the retina samples by using a lactate (0–50 nmol) standard curve.

#### Glycerol gradient centrifugation

Freshly harvested retinas were homogenized in 50 mM Tris-Cl buffer, pH 8.0 containing 150 mM NaCl, and 1 mM phenylmethylsulfonyl fluoride, and were then placed on top of the 15–35% glycerol step gradient^15^. We spun the gradients at 50,000 rpm for 16 h at 4 °C using an SW-60 Ti Backman centrifuge. Twenty fractions were collected, and protein concentration was determined using bicinchoninic acid (BCA) reagent according to the manufacturers’ instructions.

#### Electroretinography

Flash ERGs were recorded with the Diagnosys Espion E2 ERG system (Diagnosys, LLC, Lowell, MA). Mice were maintained in total darkness overnight and prepared for ERG recording under dim red light according to the method we previously published^12^. They were anesthetized with ketamine (80–100 mg/kg body weight) and xylazine (5 mg/kg body weight) intramuscularly. One drop of 10% (v/v) phenylephrine was applied to the cornea to dilate the pupil and one drop of 0.5% (v/v) proparacaine HCl was applied for local anesthesia. Mice were kept on a heating pad at 37 °C during recordings. A gold electrode was placed on the cornea, a reference electrode was positioned in the mouth, a ground electrode was placed on the foot, and the mice were placed inside a Ganzfeld illuminating sphere. Responses were differentially amplified, averaged, and stored. For the assessment of rod photoreceptor function (scotopic ERG), we used 0.0004, 0.004, 0.04, 0.4, 40, and 400 cd.s/m² flash intensities; to measure cone function (photopic ERG) we used 2000 cd.s/m² flash intensity. The amplitude of the a-wave was measured from the pre-stimulus baseline to the a-wave trough. The amplitude of the b-wave was measured from the trough of the a-wave to the peak of the b-wave. For the evaluation of cone function (photopic ERG), a strobe flash stimulus was presented to dilated, light-adapted mice. The amplitude of the cone b-wave was measured from the trough of the a-wave to the peak of the b-wave.

#### Preparation of tissue for paraffin sectioning using Prefer as a fixative

We prepared the sections according to the method we described previously^12^. Prefer solution (Anatech Ltd, Battle Creek, MI) was used to fix the mouse eyes for 15 min at room temperature, followed by 70% ethanol overnight. The tissue was paraffin-embedded, and 5-μm-thick sections were cut and mounted onto slides. These sections were subjected to immunohistochemistry. A Nikon Eclipse E800 microscope equipped with a digital camera was used to examine the antibody-labeled complexes. Metamorph (Universal Imaging, West Chester, PA) image analysis software was used to capture images under identical microscope and camera settings.

#### Immunoblot analysis

Immunoblot analysis was carried out according to the method we published previously^12^. Mouse retinas were homogenized in a lysis buffer containing 1% Triton X-100, 137 mM NaCl, 20 mM Tris-HCl (pH 8.0), 10% glycerol, 1 mM EGTA, 1 mM MgCl_2_, 1 mM phenylmethylsulfonyl fluoride, 0.2 mM Na_3_VO_4_, 10 µg/ml leupeptin, and 1 µg/ml aprotinin^16^. Insoluble material was removed by centrifugation at 17,000 × g for 20 min at 4 °C. The protein concentrations of the solubilized proteins were determined with the BCA reagent, following the manufacturer’s instructions (Pierce Biotechnology, Rockford, IL). Ten micrograms of protein (retina or retinal pigment epithelium (RPE) or choroid/sclera) were run on 10% SDS-PAGE, followed by protein blotting onto nitrocellulose membranes. After blocking the membranes with 5% non-fat dry milk power (Bio-Rad) or 5% bovine serum albumin (Sigma) for 60 min at room temperature, blots were incubated with anti-rhodopsin-1D4 (1:10,000), anti-PKM1 (1:1000), anti-PKM2 (1:1000), anti-PDE6β (1:1000), anti-pPDH (1:1000), anti-PDH (1:1000), anti-GAPDH (1:5000), anti-Rpe65 (1:1000), and anti-actin (1:1000) antibodies overnight at 4 °C. The blots were then washed and incubated with HRP-coupled anti-mouse, anti-rabbit, or anti-goat secondary antibodies for 60 min at room temperature. After washing, blots were developed with enhanced SuperSignal West Dura Extended Duration Substrate (Thermo Fisher Scientific, Waltham, MA) and visualized using a Kodak Imager with chemiluminescence capability. Densitometric analysis of immunoblots was performed in the linear range of detection. Absolute values were then normalized to either actin or GAPDH, and statistical analysis was carried out.

#### Isolation of RPE and choroid/sclera

We used an easy, rapid method to isolate RPE and choroid/sclera cell proteins from the mouse eye according to the method described in^17^. Mice were euthanized with CO_2_ asphyxiation. The eyes were enucleated and placed in a Petri dish containing phosphate-buffered saline (PBS) on ice. Under a microscope, scissors were used to incise the eye at the posterior margin of the limbus and lens. The cornea and iris were removed. The retina was removed from the eyecup and, with a pair of scissors, four small slits were cut into RPE/choroid/sclera to flatten the retina. Fifty microliters of RIPA lysis buffer containing protease inhibitors were taken into 1.5-ml Eppendorf tubes, the flattened eye cup was immersed and incubated on ice for 45 min, and the tube was tapped over 50 times to release the RPE, observed as brown material in the lysis buffer, from the rest of the eyecup containing choroid and sclera. After the incubation on ice for 45 min, the tubes were sonicated for 20–30 s. To the remaining eyecup, 50 µl of RIPA buffer was added and the eyecup was sonicated for 20–30 s to release the proteins. RPE and choroid/sclera samples were centrifuged for 15 min at 18,407 × g (Eppendorf centrifuge 5424 R) at 4 °C. The supernatants were collected into new tubes. The protein concentration was determined using BCA reagent according to the manufacturers’ instructions.

#### Pyruvate kinase enzyme assay

The lactate dehydrogenase (LDH) coupled enzyme assay was used to measure pyruvate kinase (PK) enzyme activity^9^. The assay was carried out in the presence of mouse retinal lysate containing an enzyme buffer mixture (50 mM Tris-HCl [pH 7.4], 100 mM KCl, 5 mM MgCl_2_, 1 mM ADP, 0.5 mM PEP, and 0.2 mM NADH [reduced form of NAD^+^]) and 8 U of LDH. The PK activity was measured spectrophotometrically by monitoring the reduction in the absorbance at 340 nm from the oxidation of NADH.

#### Quantitative Real-time PCR analysis (qRT-PCR)

To determine whether diabetes affects the gene expression of M1 and M2 isoforms of *Pkm* (pyruvate kinase), Pde6b (phosphodiesterase-6β), pde6c (cone-PDE), and PDHA1 (pyruvate dehydrogenase), qRT-PCR analysis was carried out on cDNA prepared from control and *db/db* mouse retina, employing primers described in Table 1. The gene expression was normalized to a housekeeping gene, CypA (cyclophillin).

**Table 1:** Real-time PCR primers used to measure gene expression.

Gene name	Forward Primer	Reverse Primer
*Pkm* (M2 isoform)	ATTGCCCGAGAGGCAGAGGC	ATCAAGGTACAGGCACTACACGCAT
*Pkm* (M1 isoform)	CTGGAATGAATGTGGCTCGG	TAAGCGTTGTCCAGGGTGAT
Pde6b	AGGTCTTGGTGCGCTTTCTA	CGAAGGCCTCTAGGTCAGTG
pde6c	ACTCCCGAAACTTCAAGTGG	TGGGTGTGATCTCTCCATGA
PDHA1	GGGACGTCTGTTGAGAGAGC	TGTGTCCATGGTAGCGGTAA
CypA	ACAGGTCCTGGCATCTTGTC	CATGGCTTCCACAATGTTCA

#### Statistical analysis

Statistical analysis employing the one-way analysis of variance and post hoc statistical tests using Bonferroni’s pairwise comparisons were used to determine significance between groups (*p* < *0.05*).

## Results

### Retinal morphology and functional characteristics of *db/db* mice

It has been previously reported that leptin-deficient (*ob/ob*) and leptin receptor-deficient (*db/db*) mice serve as models for human type 2 diabetes^18,19^. Mice homozygous for diabetes (*Lepr*^*db*^) become obese by 3–4 weeks of age, compared with C57BLKS/J mice (Fig. S1A). When the mice reached 10 weeks of age, we examined the retinal morphology for any abnormalities in the *db/db* mouse retina and compared them with C57BLKS/J mouse retina (Fig. S1B). Usually, in the photoreceptor outer nuclear layer of the retina in mice, the nuclei are arranged in 11–12 rows with no signs of retinal degeneration^20^. We found no difference in the retinal morphology between C57BLKS/J and *db/db* mice (Fig. S1C). All retinal architecture was well preserved in the neural retina of the *db/db* mice (Fig. S1C).

To determine visual function, ERG was employed when the mice were 10 weeks old. We found significantly reduced scotopic a-wave and scotopic b-wave amplitudes (rod function) in *db/db* mice compared with C57BLKS/J mice (Fig. S1D). There was no significant difference observed in photopic b-wave amplitude (cone function) between C57BLKS/J control and *db/db* mice (Fig. S1D). Collectively, the results of these experiments suggest that diabetes affects the function of the retina without affecting the structure of the retina in *db/db* mice.

### Reduced expression of PKM2 and Pde6β

**Figure 1:**
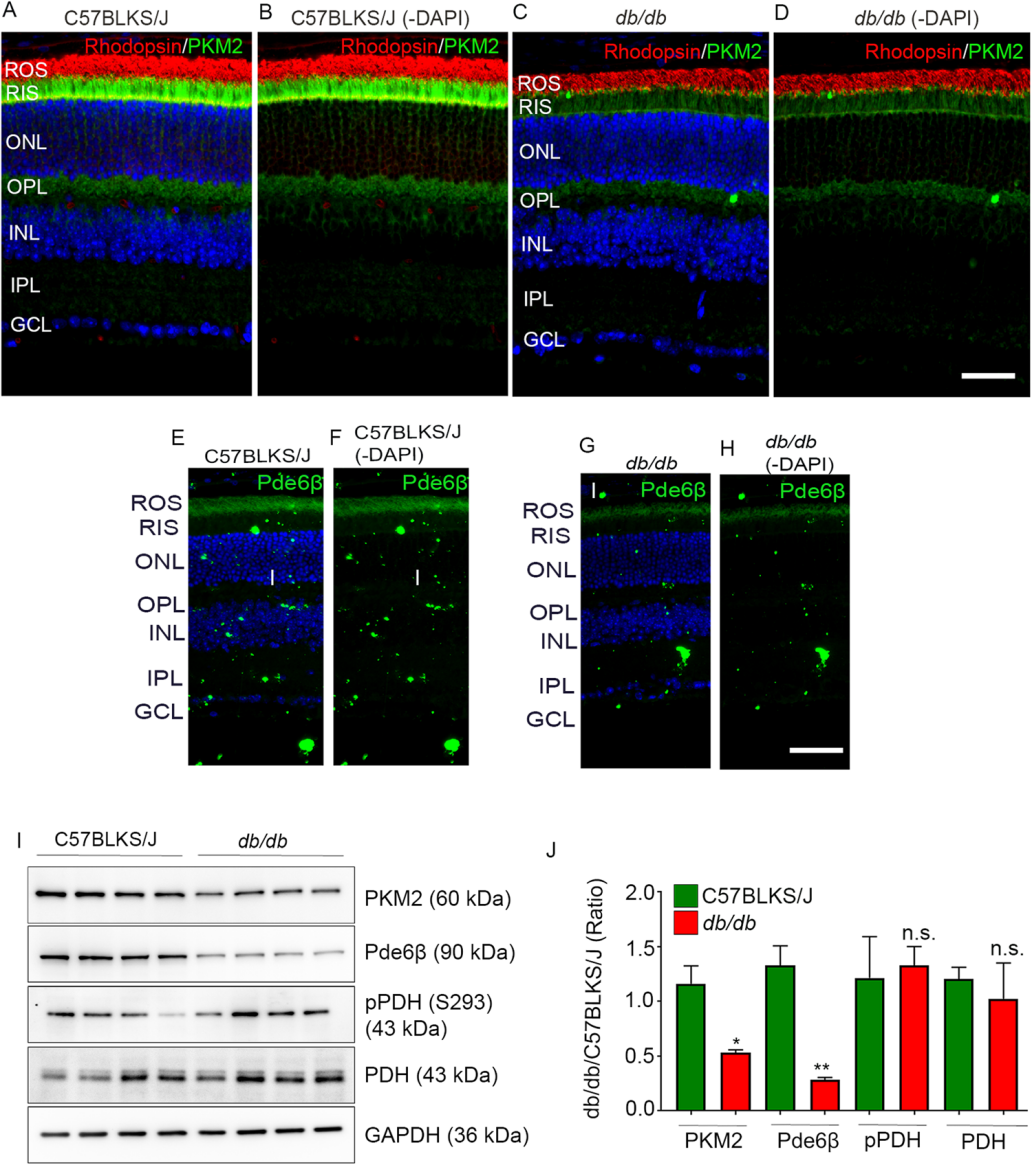
Immunofluorescence and immunoblotting analysis of PKM2 and Pde6β in *db/db* mice. Prefer-fixed sections of C57BLKS/J (**A**,**B,E,F**) and *db/db* (**C**,**D,G,H**) mouse retinas were subjected to immunofluorescence with anti-PKM2 (**A–D**, green), anti-rhodopsin (**A–D**, red), and anti-Pde6β (**E–H**) antibodies. Panels B, D, F, and H represent the images captured without DAPI staining. ROS, rod outer segments; RIS, rod inner segments; ONL, outer nuclear layer; OPL, outer plexiform layer; INL, inner nuclear layer; IPL, inner plexiform layer; GCL, ganglion cell layer. Scale bar = 50 μm. Retinal homogenates (10 µg protein) from C57BLKS/J and *db/db* mice were subjected to immunoblot analysis with anti-PKM2, anti-Pde6β, phospho-PDH, anti-PDH, and anti-GAPDH antibodies (**I**). Densitometric analysis was carried out and we normalized the protein expression to GAPDH and expressed the values as a ratio (*db/db*/ C57BLKS/J) (**J**). Data are mean + *SEM* (*n* = 4). *p < 0.005; **p < 0.0001; n.s., no significance. Full-length blots are presented in the Supplementary Information.

We recently reported that mouse rods lacking PKM2 show a reduced Pde6β expression^12^. These mice also showed significantly reduced rod function, while the structure of the retina was not significantly affected^12^. In C57BLKS/J control and *db/db* mice, we examined the expression of PKM2 and photoreceptor-specific phosphodiesterase-6β (Pde6β). The results indicated significantly reduced expression of PKM2 in *db/db* mouse retina compared with the C57BLKS/J control retina (Fig. 1A–D). Consistent with our previous observation that PKM2 transcriptionally co-activates the expression of Pde6β^12^, we observed significantly reduced expression of Pde6β in *db/db* (Fig. 1G,H) mouse retina compared with C57BLKS/J control retina (Fig. 1E,F).

An equal amount of retinal proteins from C57BLKS/J and *db/db* mouse retina probed with anti-actin antibody showed significantly increased levels of actin in *db/db* mouse retina compared with C57BLKS/J mouse retina (Fig. S2A,B). Therefore, we used GAPDH as an internal control in this study, instead of actin (Fig. 1I and Fig. S2A). We found no significant difference in the expression of rhodopsin between *db/db* and C57BLKS/J mouse retina (Fig. S2A). Retinal proteins from C57BLKS/J and *db/db* mice were immunoblotted with anti-PKM2, anti-Pde6β, anti-phospho-pyruvate dehydrogenase (pPDH-S293), anti-PDH, and anti-GAPDH antibodies (Fig. 1I). The expression levels of PKM2, Pde6β, phospho-PDH, and PDH were normalized to GAPDH (Fig. 1J). Consistent with the immunohistochemistry, the immunoblot analysis also showed a significantly reduced level of PKM2 and Pde6β in *db/db* mouse retina compared with C57BLKS/J mouse retina (Fig. 1I,J). The phosphorylation of PDH and PDH in *db/db* mouse retina is comparable to C57BLKS/J mouse retina (Fig. 1I,J). These observations suggest that protein expressions of both glycolytic and TCA cycle enzymes might be altered in *db/db* mouse retina.

### Effect of decreased expression of PKM2 protein in *db/db* mouse retina on the gene expression of *Pkm*, Pde6b, pde6c, and PDHA1

**Figure 2:**
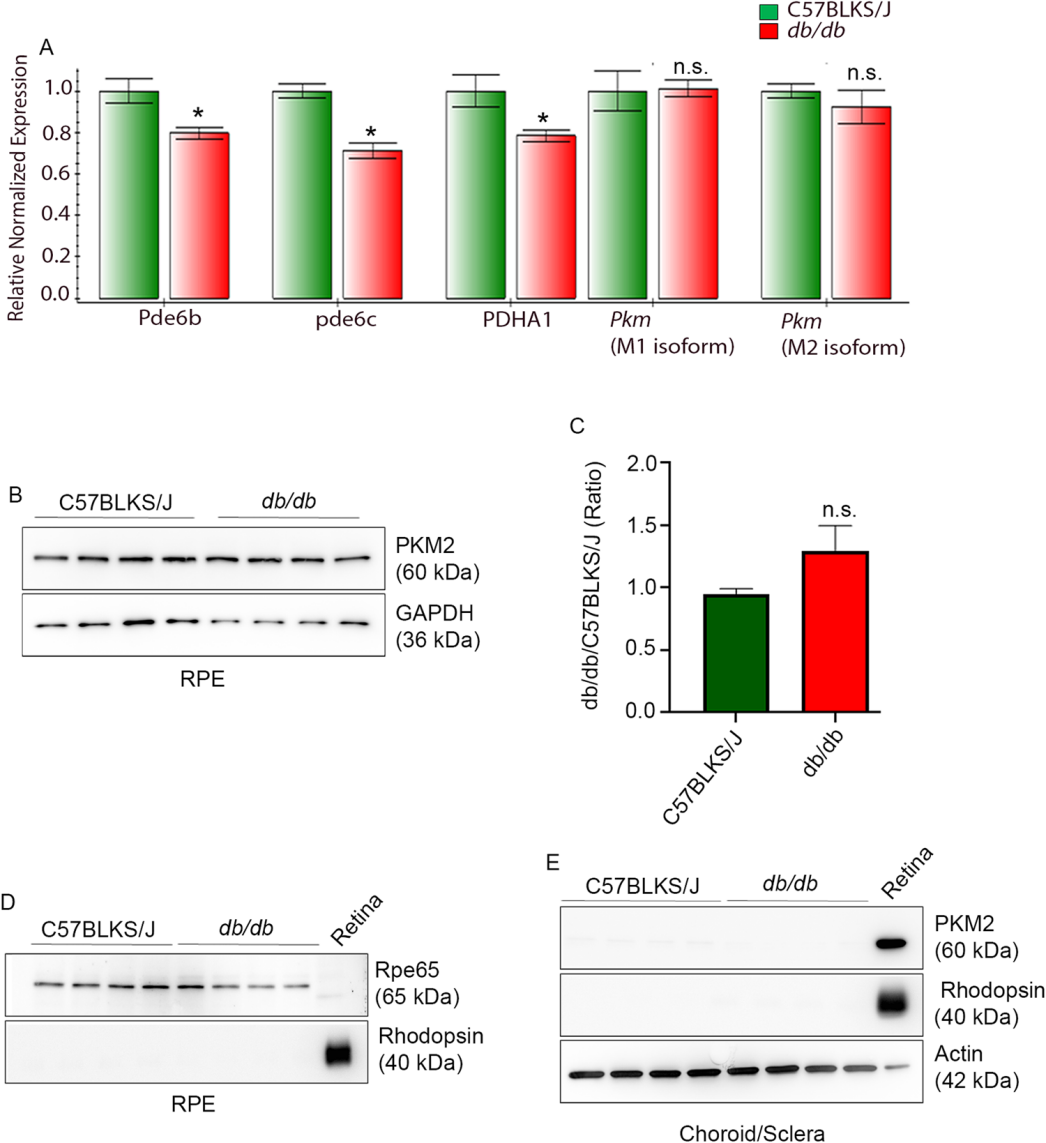
Gene expression in the retina and expression of PKM2 in retinal pigment epithelium (RPE) and choroid/sclera. Retinal mRNA from thee-independent C57BLKS/J and *db/db* mice were subjected to qRT-PCR and normalized the gene expression to cyclophyllin A (**A**). The gene expression levels were averaged for *Pkm* (M1 and M2), Pde6b, pde6c, and PDHA1. The data are mean + *SD*, (n = 3). *p < 0.05, n.s., no significance. Ten micrograms of RPE proteins were subjected to immunoblot analysis with anti-PKM2 (**B**), anti-GAPDH (**B**), anti-Rpe65 (**D**), and anti-rhodopsin (**D**) antibodies. Densitometric analysis of PKM2 was normalized to GAPDH, and we expressed the values as a ratio (*db/db*/ C57BLKS/J) (**C**). Data are mean + *SEM* (*n* = 4). n.s., no significance. Ten micrograms of choroid/sclera proteins were subjected to immunoblot analysis with anti-PKM2 (**E**), anti-rhodopsin (**E**), and anti-actin (**E**) antibodies. Full-length blots are presented in the Supplementary Information.

To determine the gene expression in *db/db* mouse retina, RNA was isolated from control and *db/db* mice, reverse transcribed to cDNA, and subjected to real-time PCR using the primers listed in Table 1. The examined genes were quantified by normalizing to CypA. We found no difference in the expression levels of *Pkm* isoforms (M1 and M2) between control and *db/db* mouse retina. However, the gene expression levels of Pde6β, pde6c, and PDHA1 were significantly lower in *db/db* mouse retina compared to the control retina (Fig. 2A). This experiment suggests that the observed reduced expression of PKM2 protein in *db/db* mouse retina could be attributable to the post-translational processing of PKM2 (degradation).

### Expression of PKM2 in retinal pigment epithelium (RPE) and choroid/sclera

Glucose from the choroidal circulation enters the RPE and is then transported to photoreceptor cells^17^. To determine whether RPE and choroid/sclera express PKM2, we prepared RPE and choroid/sclera fractions from C57BLKS/J and *db/db* mouse retina as described^21^. Proteins from the RPE fraction were immunoblotted with PKM2 and GAPDH antibodies (Fig. 2B). The results indicated that there was no significant difference in the levels of PKM2 between C57BLKS/J and *db/db* mouse RPE (Fig. 2B,C). To confirm that RPE was not contaminated with retina, we probed RPE proteins with the RPE-specific marker Rpe65 and photoreceptor marker rhodopsin antibodies. We used the mouse retina sample as a positive control for rhodopsin. We found that Rpe65 was expressed in the RPE fraction and was absent from the retina (Fig. 2D). Rhodopsin expression was present only in the retina (Fig. 2D). These experiments suggest that the RPE fraction was not contaminated with the retina. Choroid/sclera fractions from C57BLKS/J and *db/db* mice were immunoblotted with PKM2 and actin (Fig. 2E) antibodies. The results showed a complete absence of PKM2 in the choroid/sclera (Fig. 2E). We also probed the choroid/sclera with rhodopsin antibody. Rhodopsin was also absent from this fraction, suggesting that the choroid/sclera was not contaminated with the retina. Rhodopsin, which served as a positive control, was present in the retina (Fig. 2E). It is interesting to note that GAPDH expression was very low to undetectable in the choroid/sclera fraction; hence, we probed these immunoblots with anti-actin antibody as an internal control (Fig. 2E).

**Figure 3:**
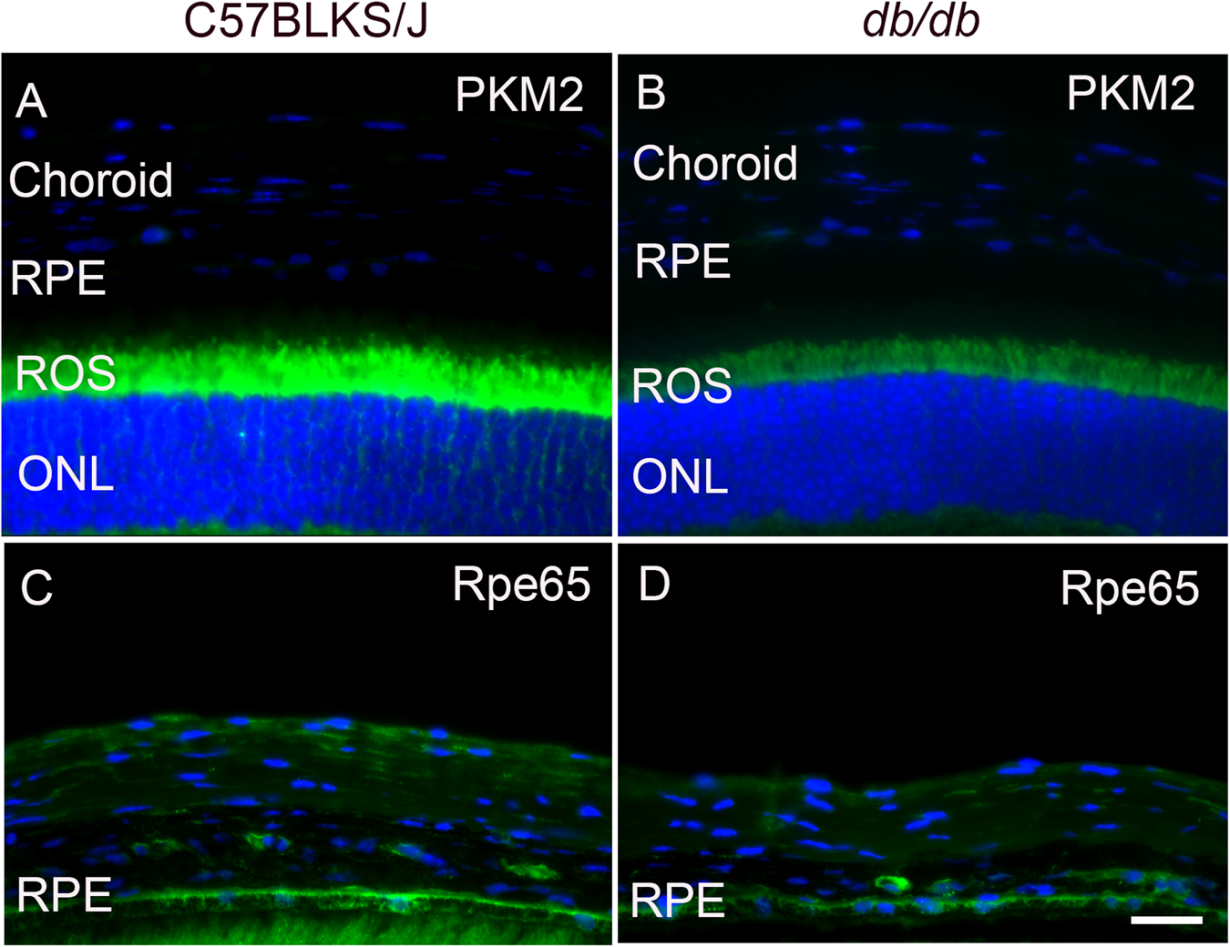
Expression of PKM2 in RPE and choroid/sclera in *db/db* mouse retina. Prefer-fixed sections of C57BLKS/J (**A,C**) and *db/db* (**B,D**) mouse retina were subjected to immunofluorescence with anti-PKM2 (**A,B**) and anti-Rpe65 (**C,D**) antibodies. RPE, retinal pigment epithelium; ROS, rod outer segments; ONL, outer nuclear layer. Scale bar = 20 μm.

Retina sections from C57BLKS/J and *db/db* mice were subjected to immunohistochemistry with anti-PKM2 and anti-Rpe65 antibody. We imaged the retina sections from the outer nuclear layer up to the choroid. At this magnification, we did not observe any detectable staining of PKM2 in the RPE and choroid region in C57BLKS/J or *db/db* mouse retina (Fig. 3A,B). The Rpe65 protein staining can be seen in the RPE of both groups (Fig. 3C,D). At this magnification, we could observe that PKM2 staining in the rod inner segments of *db/db* mouse retina is lower than the PKM2 staining in the inner segments of C57BLKS/J mouse retina (Fig. 3A,B). However, the immunoblot analysis showed the expression of PKM2 in RPE, suggesting that the PKM2 epitope in the RPE could be more accessible under denaturation conditions.

### PKM2 expression in STZ-induced diabetes

Our studies on *db/db* mouse retina showed a significant decrease in the expression of PKM2. The *db/db* mouse model is a model of obesity-associated type 2 diabetes. We examined the expression of PKM2 and PKM1 in a commonly used 6-week-old streptozotocin-induced diabetic mouse model. The results indicated that there was no significant difference in the expression of either PKM2 or PKM1 between control C57Bl/6 and STZ-induced diabetic mouse retinas (Fig. S3A,B). Retinal proteins prepared from C57Bl/6 and STZ-induced mice were immunoblotted with anti-PKM1, anti-PKM2, Pde6β, pPDH, PDH, and anti-GAPDH antibodies. Consistent with the immunohistochemistry findings, there was no significant difference in the levels of PKM1 and PKM2 between C57Bl/6 and STZ-induced diabetic mouse retinas (Fig. S3C,D). There was no significant difference in the expression of Pde6β between C57Bl/6 and STZ-induced diabetic mouse retinas (Fig. S3E,F); however, there was increased phosphorylation of PDH and PDH protein expression in STZ-induced diabetic mouse retinas compared with C57Bl/6 mouse retinas (Fig. S3E,F).

### Pyruvate kinase and lactate levels in *db/db* mouse retina

**Figure 4:**
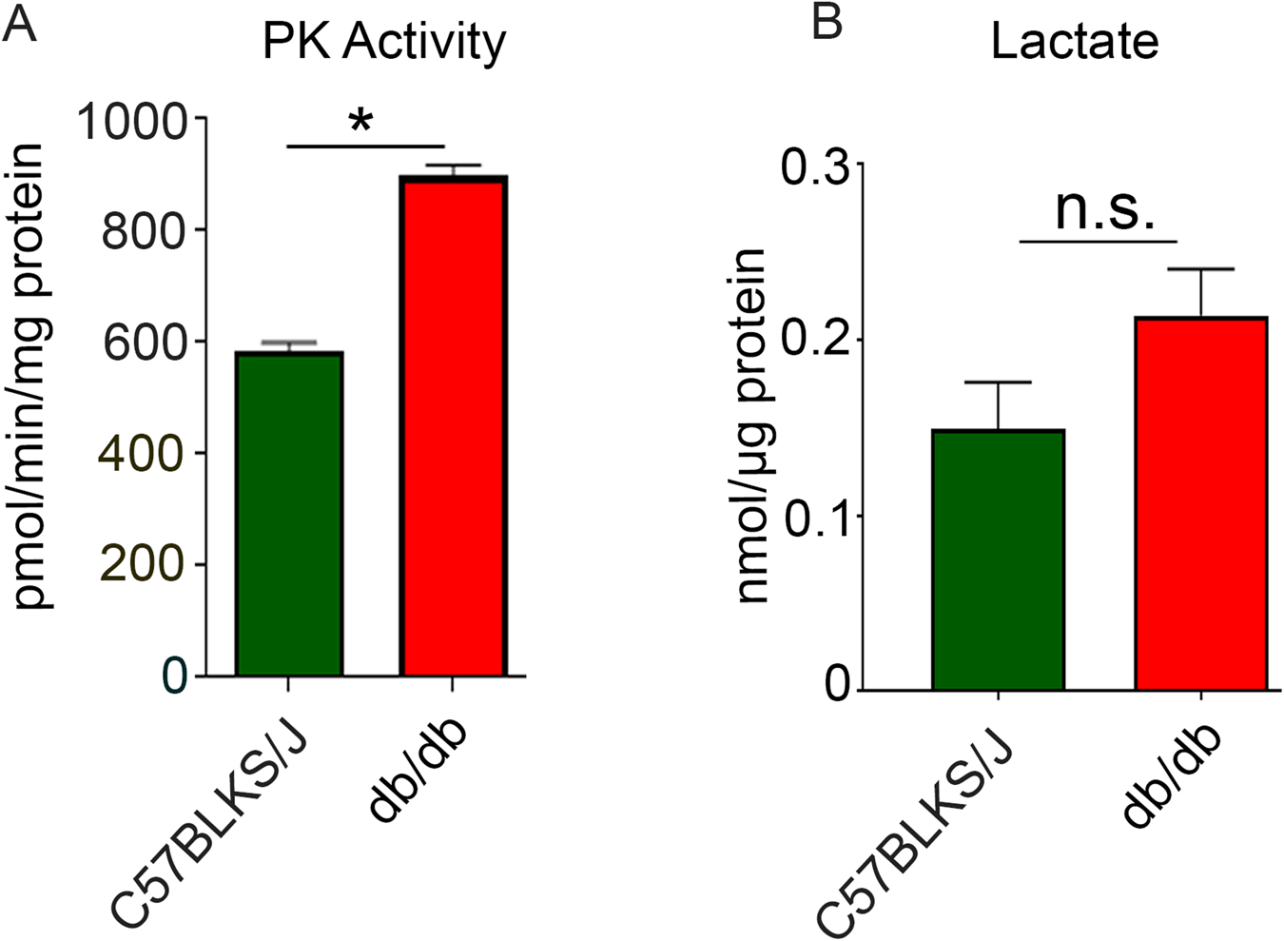
Levels of pyruvate kinase and lactate in *db/db* mice. Pyruvate kinase activity (PK) was measured with an LDH-coupled enzyme assay in the presence of 0.5 mM PEP (**A**). Lactate levels were measured using the lactate oxidase method (**B**). Data are mean + *SEM* (*n* = 6). *p < 0.0001; n.s., no significance.

To determine the effect of decreased PKM2 expression in *db/db* mouse retinas on overall pyruvate kinase (PK) activity, we measured PK activity and found that PK activity was significantly higher in *db/db* mouse retinas than in C57BLKS/J mouse retinas (Fig. 4A). We measured lactate^22^, the end product of the Warburg effect, and found increased lactate in *db/db* mouse retinas compared with C57BLKS/J mouse retinas (Fig. 4B). However, the difference was not statistically significant.

### The state of PKM2 in C57BLKS/J and *db/db* mouse retina

**Figure 5:**
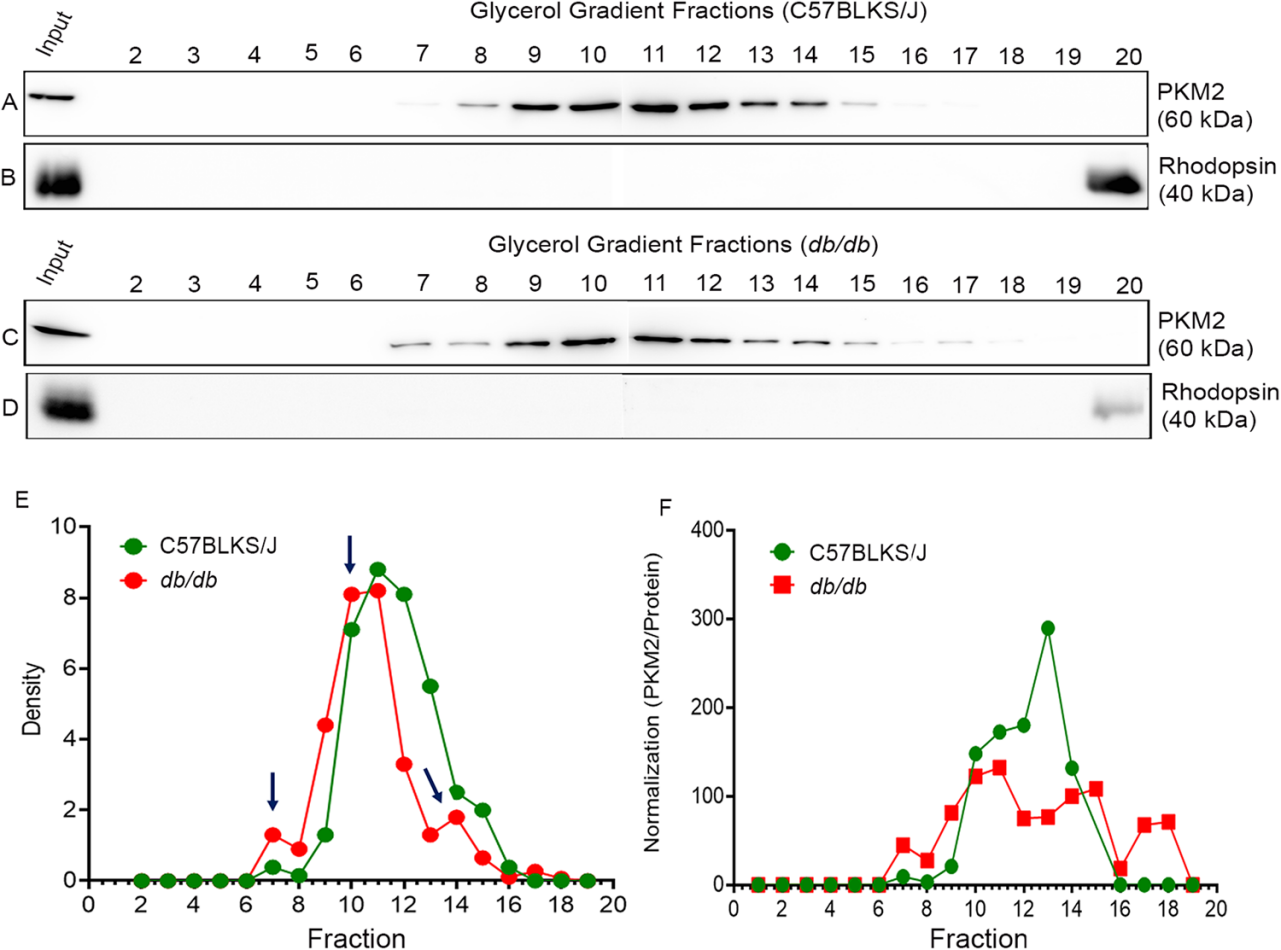
Glycerol density gradient centrifugation to determine PKM2 oligomers. Retina lysates from C57BLKS/J and *db/db* mouse retina were loaded on top of the 10–35% glycerol gradient, and the samples were spun at 50,000 rpm for 16 h at 4 °C. Fractions were collected and subjected to immunoblot analysis with anti-PKM2 (**A,C**) and anti-rhodopsin (**B,D**) antibodies. PKM2 density was calculated and plotted the graph against each fraction (**E**) and normalized the density of each fraction with each fraction protein (**F**). Arrow indicates PKM2 peaks in *db/db* mouse retina. We collected 20 fractions from the glycerol gradients, cannot accommodate in a single gel. Hence, we run on two gel (10 samples on each gel) and joined them together. Full-length blots are presented in the Supplementary Information.

Depending on the posttranslational modification and allosteric effector regulation, PKM2 exists as a monomer (60 kDa), dimer (120 kDa), and tetramer (240 kDa)^13^. The tetrameric form of PKM2 promotes oxidative phosphorylation, whereas the dimeric form of PKM2 promotes Warburg effect^23^. In the present study, we found significantly increased pyruvate kinase activity in *db/db* mouse retinas compared with C57BLKS/J mouse retinas (Fig. 4A). To determine whether this activity is contributed by an increased tetrameric form of PKM2 in *db/db* mouse retina, we fractionated the control and *db/db* mouse retina lysates through glycerol density gradient centrifugation, followed by immunoblotting analysis with anti-PKM2 (Fig. 5A,C) and anti-rhodopsin antibody (Fig. 5B,D). Protein determination revealed the elution of protein as a single peak (fractions 6–14) in both control and *db/db* mouse retina (Fig. S4). The rhodopsin immunoreactivity showed a single enriched fraction (fraction 20, the final fraction at the bottom), suggesting that membrane proteins are sedimented under the centrifugal speed used in this study (Fig. 5B,D). When we calculated the density of each band and plotted the graph against each fraction indicate that the PKM2 elution pattern of *db/db* mouse retina is distinct from the control retina (Fig. 5E). The elution profile shows that PKM2 in *db/db* mouse retina appears several peaks and the peak fraction of PKM2 in *db/db* mouse retina comes earlier than the control retina (Fig. 5E). When we normalized PKM2 density in each fraction with each fraction protein, it appears the PKM2 expression levels are lower in db/db mouse retina compared to the control retina (Fig. 5F). Collectively these observations suggest that PKM2 in *db/db* mouse retina may appear to exist in higher-order structures when compared to control retina that might partly explain why *db/db* mouse retina has a higher pyruvate kinase activity.

### Expression of PKM1 *db/db* mouse retina

**Figure 6:**
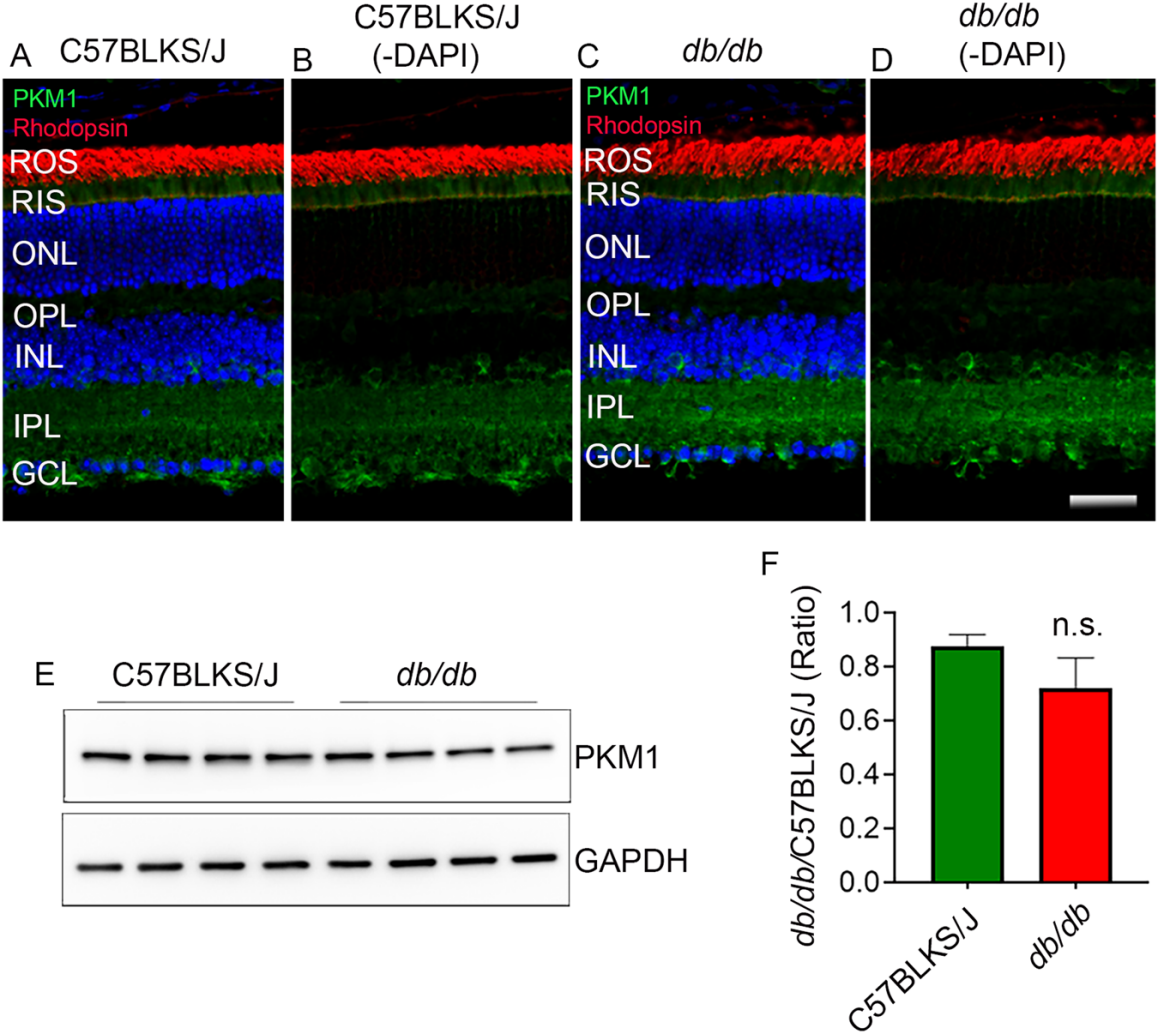
Expression of PKM1 in C57BLKS/J and *db/db* mouse retina. Prefer-fixed sections of C57BLKS/J (**A**,**B**) and *db/db* (**C,D**) mouse retinas were subjected to immunofluorescence with anti-PKM1 (**A–D**, green) and anti-rhodopsin (**A–D**, red) antibodies. Panels B, and D represent the images captured without DAPI staining. ROS, rod outer segments; RIS, rod inner segments; ONL, outer nuclear layer; OPL, outer plexiform layer; INL, inner nuclear layer; IPL, inner plexiform layer; GCL, ganglion cell layer. Scale bar = 50 μm. Ten micrograms of retinal proteins were subjected to immunoblot analysis with anti-PKM1, and anti-GAPDH antibodies (**E**). Densitometric analysis of PKM1 was normalized to GAPDH, and we expressed the values as a ratio (*db/db*/ C57BLKS/J) (**F**). Data are mean + *SEM* (*n* = 4). n.s., no significance. Full-length blots are presented in the Supplementary Information.

We previously reported that PKM1 expression is predominantly observed in the inner plexiform and ganglion cell layer, with some staining in the rod inner segments^9^. To determine whether obesity-induced diabetes has any effect on the expression of PKM1, we immunostained the C57BLKS/J and *db/db* mouse retina sections with anti-PKM1 and anti-rhodopsin antibodies (Fig. 6A–D). The immunohistochemistry results indicated that there were no detectable changes in the expression of PKM1 between C57BLKS/J and *db/db* mouse retina (Fig. 6A–D). The rhodopsin staining also appeared to be normal in *db/db* mouse retina, and the expression of PKM1 was comparable to that of C57BLKS/J mice (Fig. 6A–D). Consistent with these observations, the immunoblot analysis carried out with the anti-PKM1 antibody further shows that there was no significant difference in the protein expression levels between *db/db* and C57BLKS/J retina (Fig. 6E,F).

### Altered levels of NAD+, NADH, NADP+, and NADPH in *db/db* mouse retina

**Figure 7:**
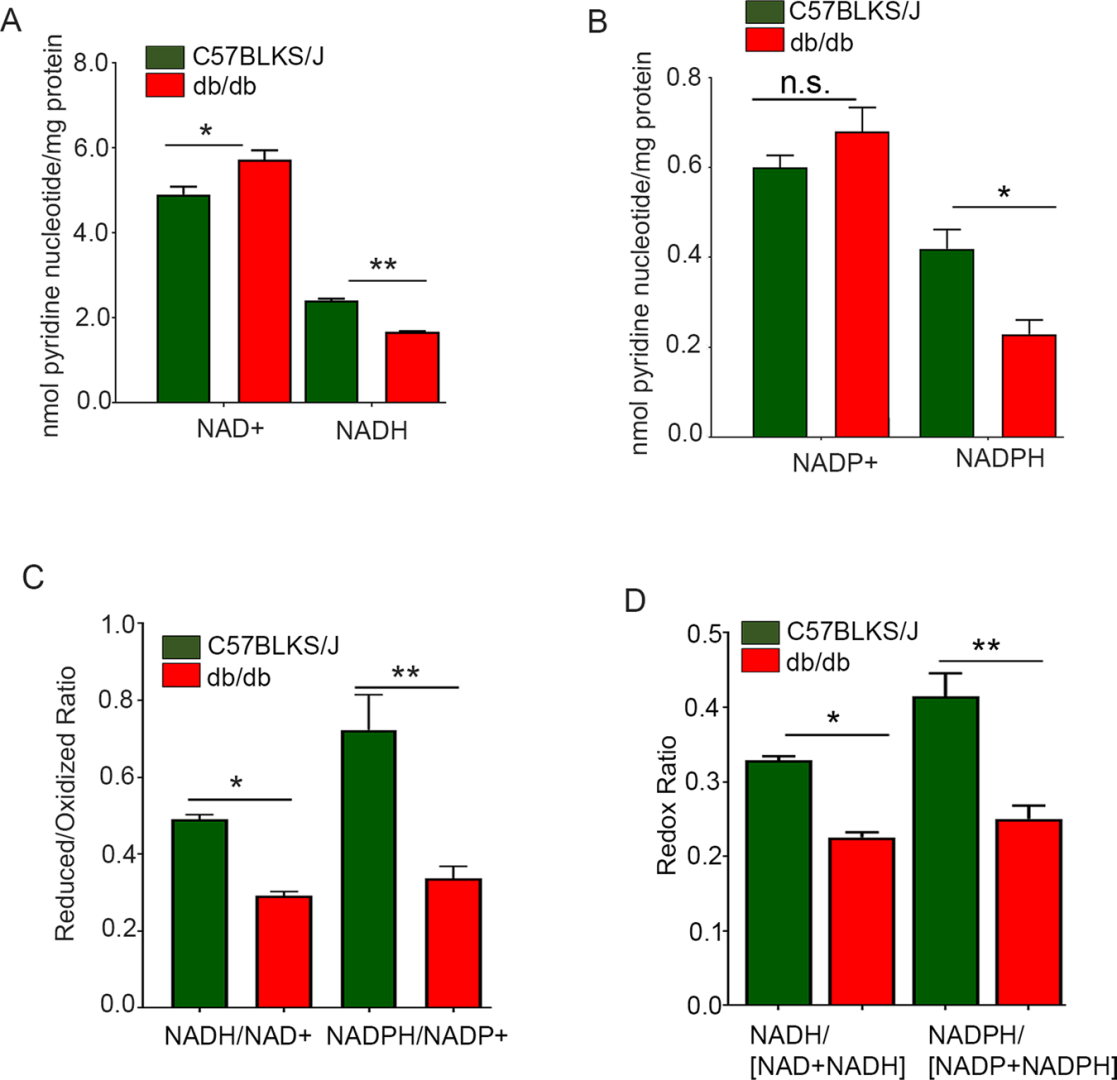
Oxidized and reduced levels of pyridine nucleotides and their ratios in *db/db* mouse retina. We calculated retinal pyridine nucleotides from C57BLKS/J and *db/db* mouse retina from the standard curve carried out with different concentrations of pyridine nucleotides (Fig. S3). NAD+ (**A**), NADH (**A**), NADP+ (**B**), and NADPH (**B**) levels were measured from C57BLKS/J and *db/db* mouse retina. Data were expressed for each co-factor. Ratios and redox states of these molecules were also calculated (**C**,**D**). Data are mean + *SEM*, (*n* = 5). Significance for panel A (*p < 0.028; **p < 0.01); panel B (*p < 0.01, n.s., no significance); panel C (*p < 0.0001; **p < 0.0072) and panel D (*p < 0.0001; **p < 0.0037).

We examined the levels of NAD+, NADH, NADP+, and NADPH in *db/db* and C57BLKS/J mouse retinas. We standardized the method with different known concentrations of pyridine nucleotides (Fig. S5) and examined different volumes of retina samples collected from C57BLKS/J (Fig. S5B) and *db/db* (Fig. S5C) mouse retinas. NAD+ and NADP+ levels were higher in *db/db* mouse retinas than in C57BLKS/J mouse retinas (Fig. 7A,B). The NADH and NADPH levels were significantly lower in *db/db* mouse retinas than in C57BLKS/J mouse retinas (Fig. 7A,B). In the diabetic retina, there was a decrease in the ratios of reduced/oxidized NADH/NAD+ and NADPH/NADP+ compared with the control retina, suggesting less availability of NADH and NADPH in diabetic mouse retina (Fig. 7C). Consistent with these observations, we found significantly reduced redox potential (NADH/[NAD^+^ + NADH] and (NADPH/[NADP^+^ + NADPH]) in *db/db* mouse retina compared with controls (Fig. 7D).

## Discussion

Previously published studies show that PKM2 performs two functions. As a metabolic enzyme, PKM2 converts phosphoenolpyruvate (PEP) to pyruvate and promotes the activation of the pentose phosphate pathway (PPP)^10^. It functions in a non-metabolic manner as a transcriptional co-activator in the synthesis of various enzymes and transporters related to energy metabolism^10^. Studies from our laboratory showed that the ablation of PKM2 in rod photoreceptors resulted in almost 98 percent loss of PKM2 in rod photoreceptor cells^12^. We found that loss of PKM2 resulted in a significant loss of rod function^12^.

We reported that the expression of Pde6β was significantly lower in mice with rods lacking PKM2 than in wild-type mice^12^. Our previous studies also showed that Pde6β promoter activity is regulated by PKM2 *in vitro*^12^. The reduced rod function in PKM2 deleted rods can be explained by reduced levels of Pde6β^12^. A decreased visual function has also been reported in animal models of diabetes^4,5^, including the *db/db* mouse model^24^. Very recently, PKM2 activation was reported to protect against the progression of diabetic glomerular pathology and mitochondrial dysfunction^13^. These studies led to the hypothesis that reduced vision loss in diabetes could be due to decreased levels of PKM2 in diabetes.

In the present study, we found a decreased expression of PKM2, but not PKM1, in *db/db* mouse retina. Under these conditions, we found significantly increased pyruvate kinase activity in this mouse retina. The difference between PKM1 and PKM2 is only 22 amino acids, but the functions mediated by PKM2 are unique, as it induces metabolic reprogramming and the Warburg effect in cancer cells^23^. The isoform PKM1 forms a constitutively stable active tetramer^23^, whereas PKM2 tetrameric configuration is regulated by numerous modifications, such as phosphorylation, acetylation, oxidation and also controlling the activity by an allosteric activator, fructose 1–6- bisphosphate (FBP)^23^. PKM2 undergoes posttranslational modifications, and it exists as a dimer with low PKM2 activity whereas FBP binding results in a tetrameric form with high PKM2 activity. These forms can be easily separated on a density gradient centrifugation^23^. In diabetic kidney glomeruli, tetramer, dimer, and monomers of PKM2 have been reported^13^. Our studies on glycerol gradient centrifugation show that PKM2 in *db/db* mouse retina may appear to exist in higher-order structures when compared to control retina that might partly explain why *db/db* mouse retina has a higher pyruvate kinase activity.

One interesting finding was that the PKM2, but not PKM1, protein level was decreased in *db/db* mouse retina; however, the pyruvate kinase activity was significantly increased. We know from the literature that PKM2 transcriptionally represses the expression of PKM1^25^. We^12,26^ and others^27,28^ have shown that the deletion of PKM2 upregulates the expression of PKM1. Our studies suggest that decreased expression of PKM2 may relieve the inhibition constrains on PKM1, which might result in increased pyruvate kinase activity. In STZ mice, there was no change in either PKM2 or PKM1 expression, suggesting that an altered PKM2/PKM1 ratio might be critical for both metabolic and non-metabolic functions. Further studies are needed to establish the relationship in health and disease.

It was previously reported that diabetes increases glycolysis and β-oxidation metabolites in the *db/db* mouse retina^29^. In this study, the authors used a systemic approach with transcriptomics, metabolomics, and metabolic flux analysis, and observed tissue-specific differences with increased glucose and fatty acid metabolism in the kidney, a moderate increase in the retina, and a decrease in the nerve of *db/db* mice^29^. Increased levels of long-chain acylcarnitines and glycolytic metabolites have been observed in 6-month-old *db/db* mouse retina^29^. However, they did not observe a significant difference in the level of acetyl CoA. Significantly increased levels of malate and succinate were present, but the citrate level was significantly decreased in *db/db* mouse retina^29^, suggesting an increase in energy metabolism. The pattern was not uniformly reflected by the metabolites in the TCA cycle.

In the present study, there was no change in the retinal PKM2 expression in animals with streptozotocin-induced diabetes. The STZ-induced diabetes model represents type 1 diabetes^30^. The *db/db* mouse serves as a model for human type 2 diabetes^18,19^. In this model, we found reduced retinal PKM2 expression, reduced expression of Pde6β, and reduced rod function. We also observed decreased gene expression of rod (Pde6b) cone PDE (pde6c), but there was no decrease in the gene expression of M1 and M2 isoforms of *Pkm* in *db/db* mouse retina compared with control retina. In diabetic nephropathy (DN), PKM2 expression and activity are upregulated^13^. The knockdown of PKM2 in immortalized podocytes has been shown to produce increased levels of toxic glucose metabolites, mitochondrial dysfunction, and increased cell death^13^. Pharmacological activation of PKM2 in diabetic podocyte-specific PKM2 knockdown mice have shown to reverse the albuminuria and glomerular diabetic nephropathy pathology^13^. These studies provide evidence that PKM2 activation may protect DN pathology. In an independent study, the gene expression of PKM2 has not been altered in diabetic kidney^29^.

A 2013 study of human breast cancer cells and the patients’ primary breast cancer tissue showed significant expression of Pde6β, but no other photoreceptor-specific genes^31^. However, that study did not investigate PKM2 expression, while others reported PKM2 overexpression in breast tumors^31^. We previously reported that Pde6β promoter activity assays indicated that PKM2 may regulate PDE6β expression *in vivo*^12^. PKM2 has been shown to act as a transcriptional co-activator for the induction of glycolytic genes, including PKM2^10^. The reduced PKM2 protein expression may decrease transcriptional co-activator activity, which might result in reduced expression of Pde6β. In the present study, we did not observe any decrease of PKM2 gene expression, but PKM2 protein was decreased, suggesting an increased protein half-life. Such a possibility cannot be ruled out in the *db/db* mouse retina. The decreased PDE6β expression is attributable to decreased PKM2 transcriptional co-activator activity. PKM2 may bind to pde6c and PDHA1 promoter/enhancer regions and regulate their expression, and in this study, we observed an altered gene expression of cone PDE (pde6c) and pyruvate dehydrogenase (PDHA1) in *db/db* mouse retina. Further studies are needed to confirm these observations.

Nicotinamide adenine dinucleotide (NAD+), the reduced form of nicotinamide adenine dinucleotide (NADH), nicotinamide adenine dinucleotide phosphate (NADP+), and the reduced form of nicotinamide adenine dinucleotide phosphate (NADPH) are important biomolecules known to be involved in antioxidant metabolism, energy metabolism, and reductive biosynthetic metabolism^32–34^. The biomolecule NADP+ is almost identical to NAD+, except for an extra 2′ phosphate on the adenosine ribose moieties of NADP+. The substrate oxidation catalyzed by the enzymes predominantly uses both NAD + and NADH, whereas enzymes that catalyze substrate reduction use both NADP+ and NADPH^34^. In the *db/db* mouse model, there was a decrease in the ratios of reduced/oxidized NADH/NAD and NADPH/NADP compared with those of control retina, suggesting less availability of NADH and NADPH in the diabetic mouse retina. Consistent with these observations, we found significantly reduced redox potential (NADH/[NAD^+^ + NADH] and (NADPH/[NADP^+^ + NADPH]) in *db/db* mouse retina. Furthermore, the increased pyruvate kinase activity suggests a decreased anabolic activity in the retina.

In summary, we observed changes in both metabolic and non-metabolic functions. The metabolic functions, including increased PK activity and lactate levels and a decreased ratio of reduced/oxidized redox, decreased mitochondrial oxidation. The non-metabolic functions included decreased PKM2 expression, which might affect the transcriptional co-activator ability of PKM2, resulting in reduced Pde6β expression, which produces decreased rod function in diabetes. Overall, this study provides new knowledge to the field that PKM2 could serve as a new therapeutic target for obesity-induced diabetic retinopathy.

## Future studies and limitations

In the future, we will study the post-transcriptional regulation of PKM2 in *db/db* mice and the possible transcriptional activity of PKM2 and its interaction with Pde6c and PDH promoter/enhancer regions. In *db/db* mouse retina, we found decreased PDH gene expression whereas the protein expression appears to be normal. These observations suggest some post-transcriptional or post-translational events might play a role in PDH gene expression in diabetes, will study this aspect in the future. In the future, we will overexpress PKM2 protein in the retina of *db/db* mice using AAV-viral constructs and examine the improvement of ERG, PDE6β level and, metabolic functions. These studies are long term and are beyond the scope of the current study.

## Supplementary information

Supplementary Information.

Supplementary Information2.
