# Supplementary material for: Metabolic and Non-metabolic Roles of Pyruvate Kinase M2 Isoform in Diabetic Retinopathy
Source: Sci Rep. 2020 May 4;10:7456. doi: 10.1038/s41598-020-64487-2 (PMC7198623; doi:10.1038/s41598-020-64487-2)
Supplement: Supplementary file 1 — Supplementary Information. [file 41598_2020_64487_MOESM1_ESM.pdf]

Metabolic and Non-metabolic Roles of Pyruvate Kinase M2 Isoform in Diabetic Retinopathy

Ammaji Rajala, Krutik Soni and Raju V.S. Rajala

Figure 1

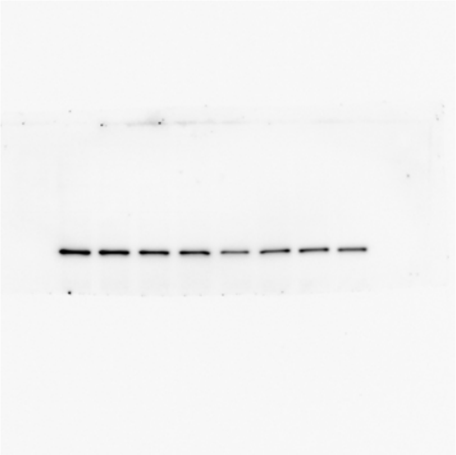

Full-length blot of Figure 1 (I) in the main text (PKM2).

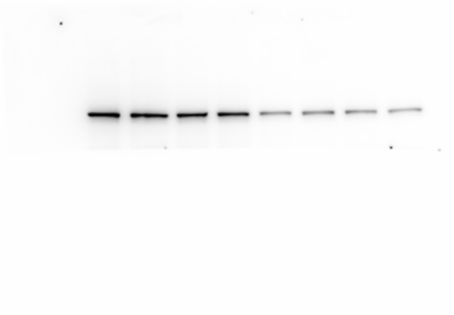

Full-length blot of Figure 1 (I) in the main text (Pde6β).

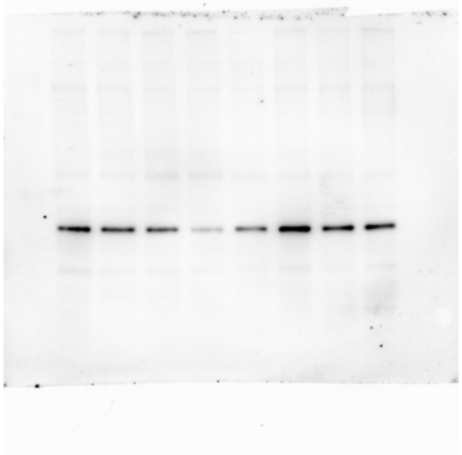

Full-length blot of Figure 1 (I) in the main text (pPDH).

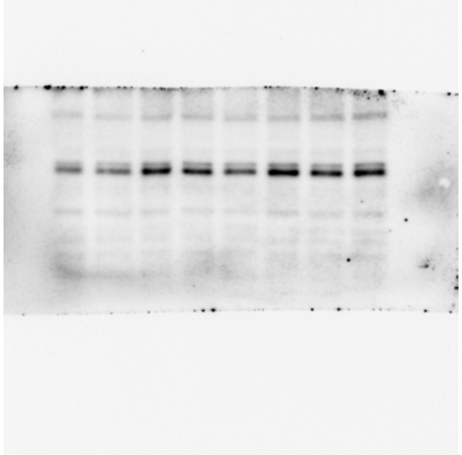

Full-length blot of Figure 1 (I) in the main text (PDH).

Full-length blot of Figure 1 (I) in the main text (GAPDH).

Figure 2

Full-length blot of Figure 2 (B) in the main text (PKM2).

Full-length blot of Figure 2 (B) in the main text (GAPDH).

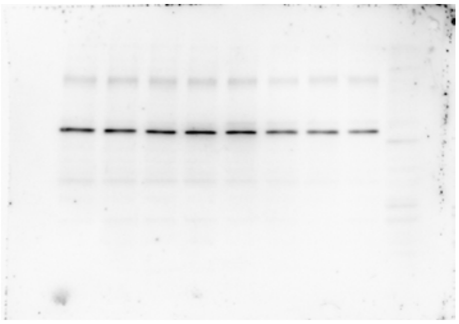

Full-length blot of Figure 2 (D) in the main text (Rpe65).

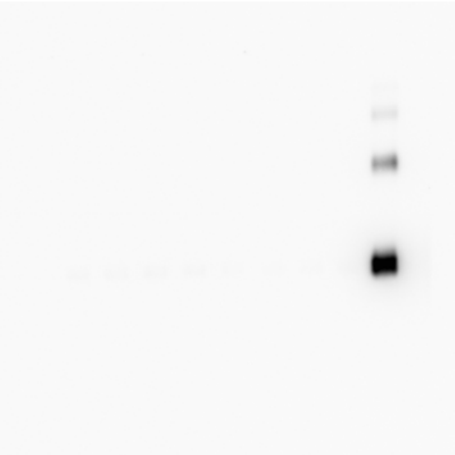

Full-length blot of Figure 2 (D) in the main text (rhodopsin).

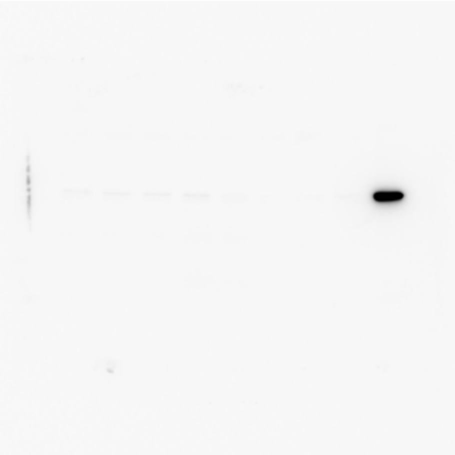

Full-length blot of Figure 2 (E) in the main text (PKM2).

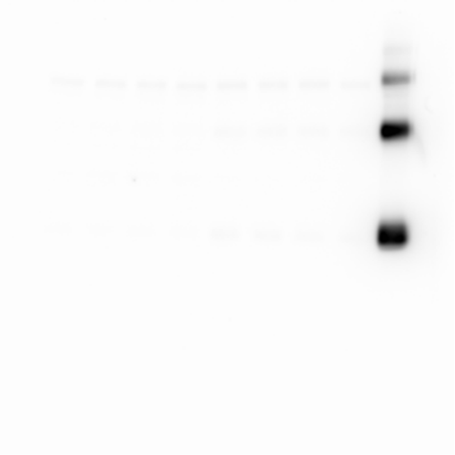

Full-length blot of Figure 2 (E) in the main text (rhodopsin).

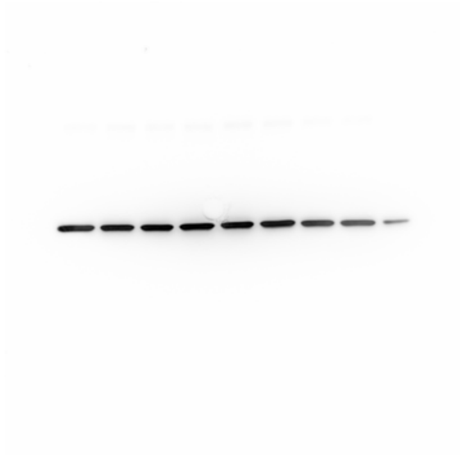

Full-length blot of Figure 2 (E) in the main text (Actin).

Figure 5

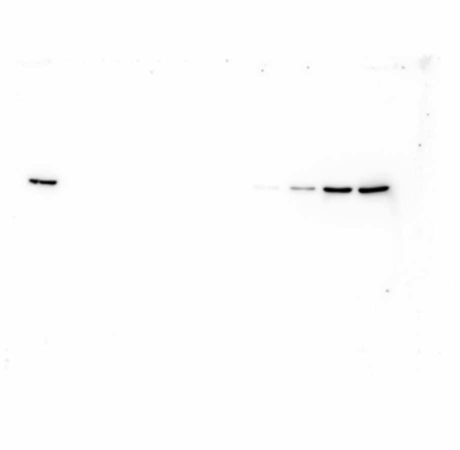

Full-length blot of Figure 5 (A) in the main text (PKM2, Fr # 1-10).

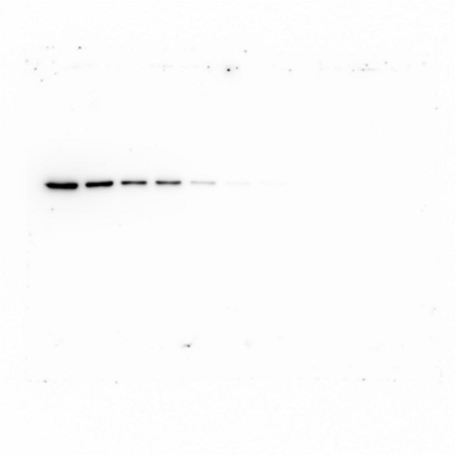

Full-length blot of Figure 5 (A) in the main text (PKM2, Fr # 11-20).

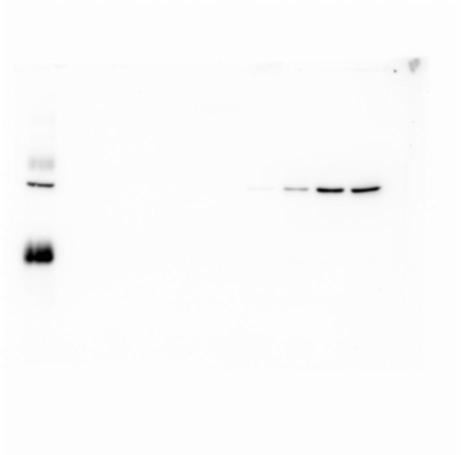

Full-length blot of Figure 5 (B) in the main text (rhodopsin, Fr # 11-20).

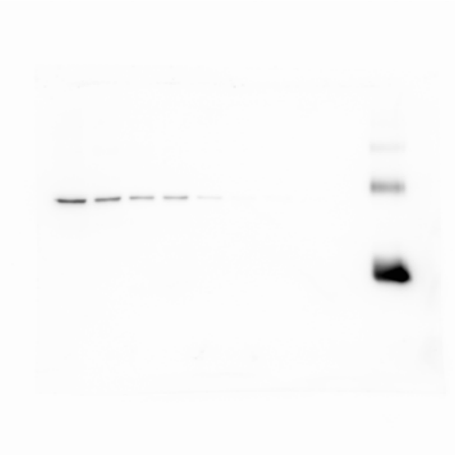

Full-length blot of Figure 5 (B) in the main text (rhodopsin, Fr #11-20).

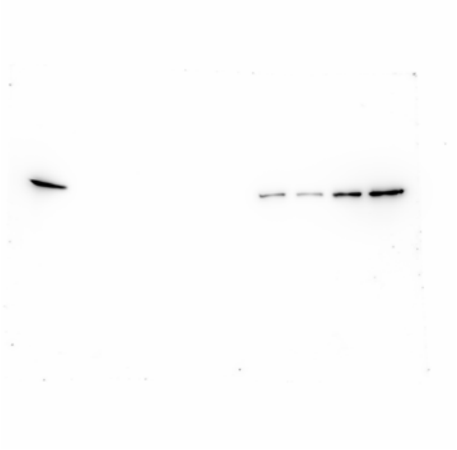

Full-length blot of Figure 5 (C) in the main text (PKM2, Fr#1-10).

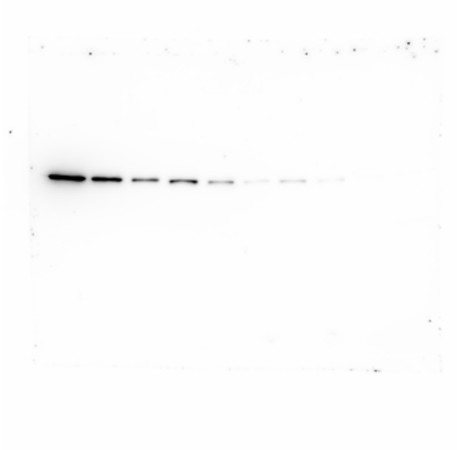

Full-length blot of Figure 5 (C) in the main text (PKM2, Fr #11-20).

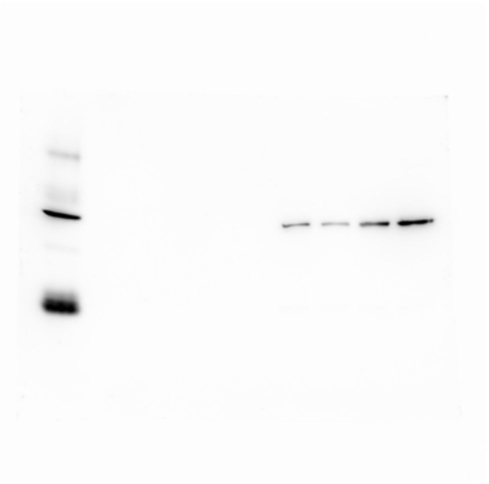

Full-length blot of Figure 5 (D) in the main text (rhodopsin, Fr #1-10).

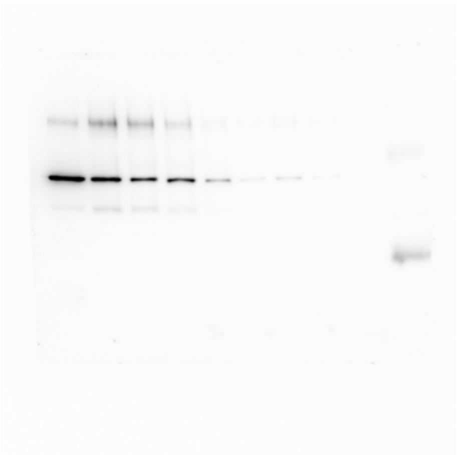

Full-length blot of Figure 5 (D) in the main text (rhodopsin, Fr #11-20).

Figure 6

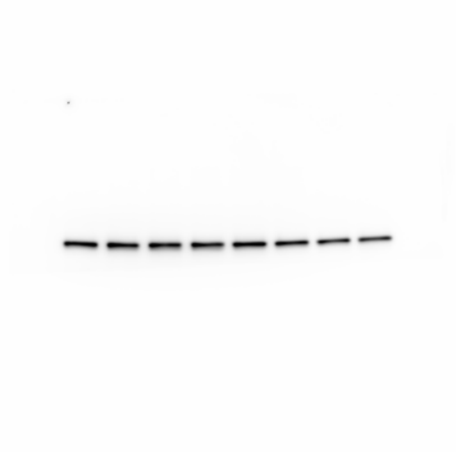

Full-length blot of Figure 6 (E) in the main text (PKM1).

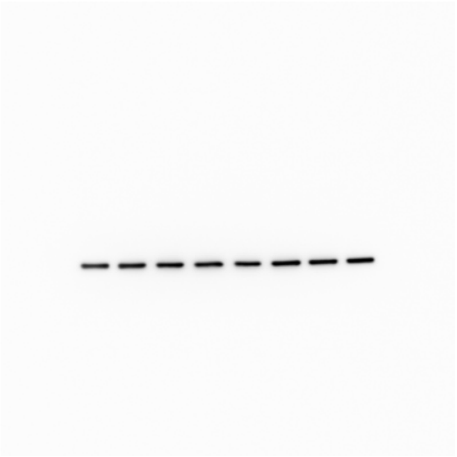

Full-length blot of Figure 6 (E) in the main text (GAPDH).

Figure S2

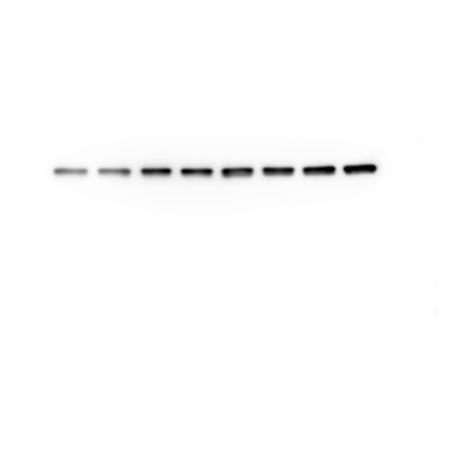

Full-length blot of Figure S2 (A) in the main text (Actin).

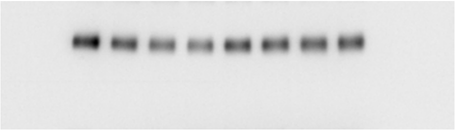

Full-length blot of Figure S2 (A) in the main text (Rhodopsin).

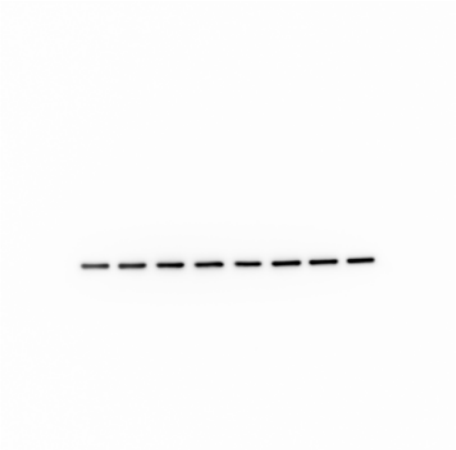

Full-length blot of Figure S2 (A) in the main text (GAPDH).

Figure S3

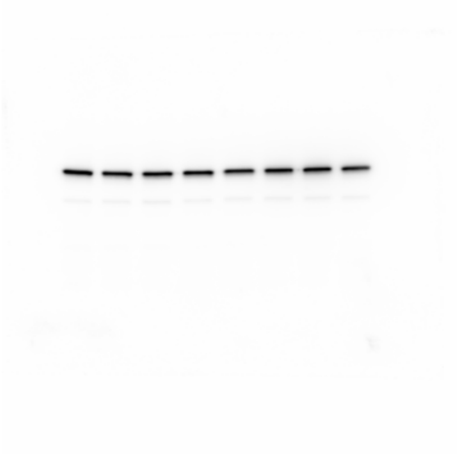

Full-length blot of Figure S3 (C) in the main text (PKM1).

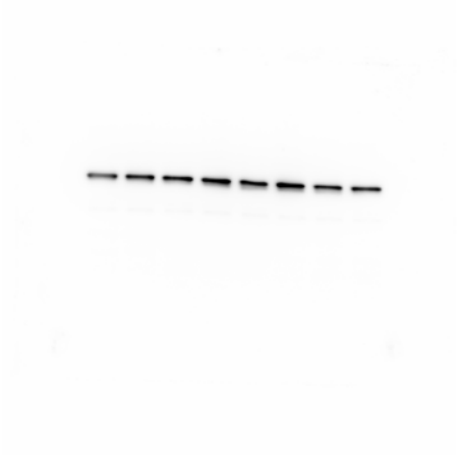

Full-length blot of Figure S3 (C) in the main text (PKM2).

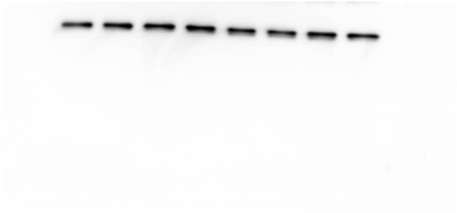

Full-length blot of Figure S3 (C) in the main text (Actin).

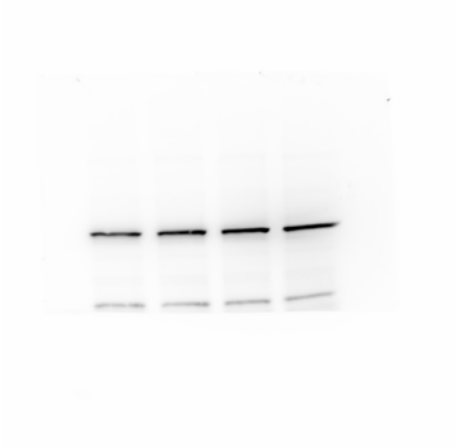

Full-length blot of Figure S3 (E) in the main text (Pde6β).

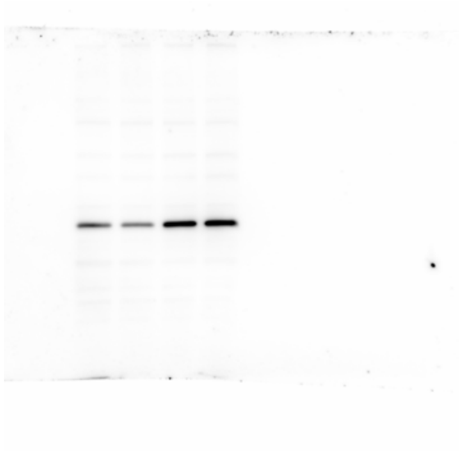

Full-length blot of Figure S3 (E) in the main text (pPDH).

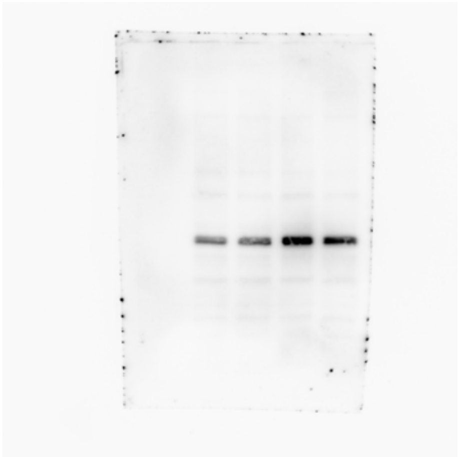

Full-length blot of Figure S3 (E) in the main text (PDH).

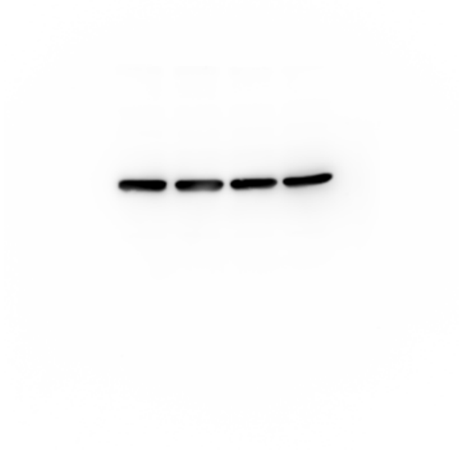

Full-length blot of Figure S3 (E) in the main text (GAPDH).
