# Supplementary material for: Metabolic and Non-metabolic Roles of Pyruvate Kinase M2 Isoform in Diabetic Retinopathy
Source: Sci Rep. 2020 May 4;10:7456. doi: 10.1038/s41598-020-64487-2 (PMC7198623; doi:10.1038/s41598-020-64487-2)
Supplement: Supplementary file 2 — Supplementary Information2. [file 41598_2020_64487_MOESM2_ESM.docx]

**
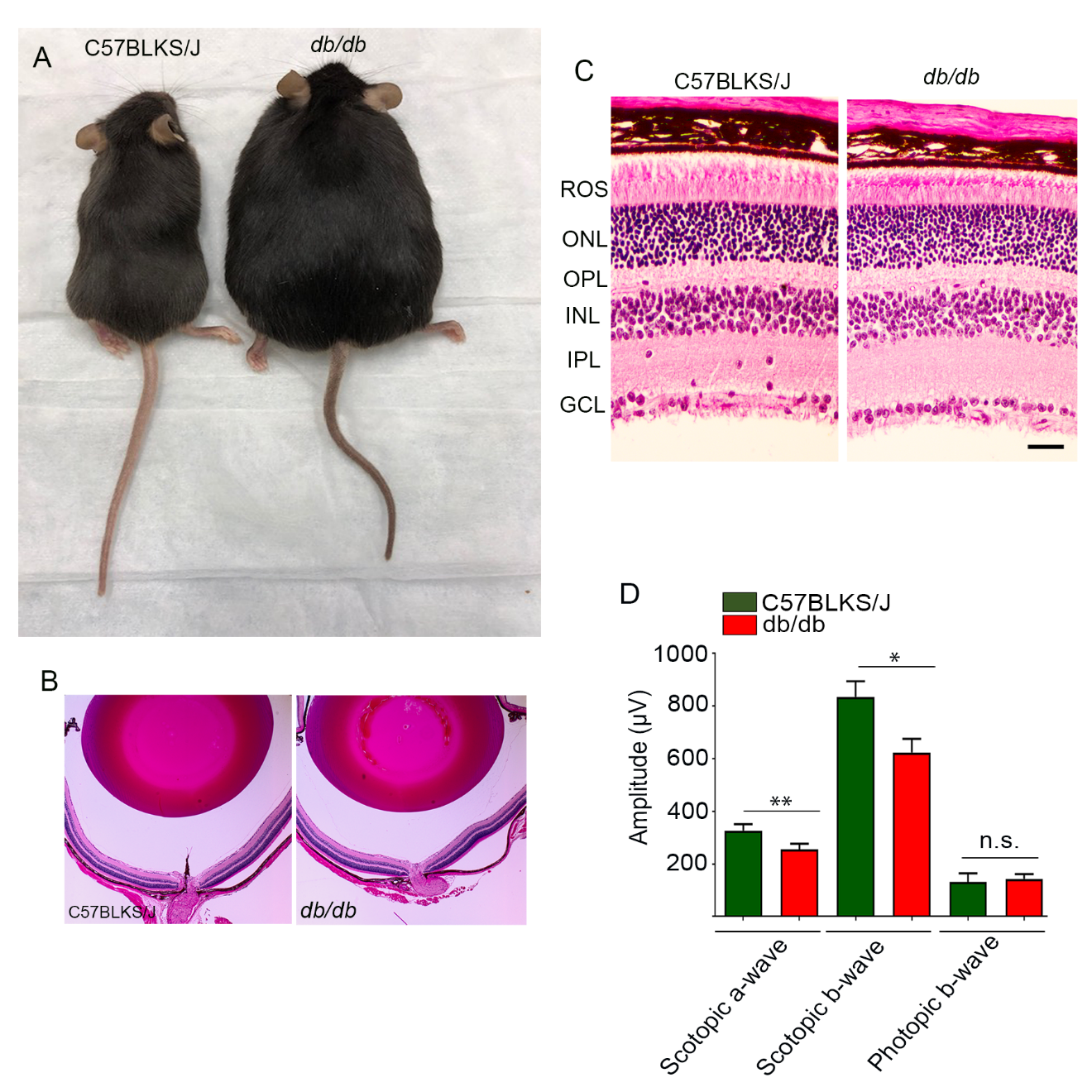
Figure S1. Morphological and functional characteristics of *db/db* mice.** Phenotypically, the *db/db* mice are obese compared with C57BLKS/J mice (**A**). Five-micrometer-thick sections of retinas from C57BLKS/J and *db/db* (**B**) mice were cut along the vertical meridian and stained with hematoxylin and eosin. A region of the retina near the optic nerve head from C57BLKS/J (and *db/db* (**C**) was enlarged for fine details. Scale bar = 50 μm. ROS, rod outer segments; ONL, outer nuclear layer; OPL, outer plexiform layer; INL, inner nuclear layer; IPL, inner plexiform layer; GCL, ganglion cell layer. Scotopic a-wave, scotopic b-wave, and photopic b-wave electroretinographic analysis of retinas from 10-week-old C57BLKS/J and *db/db* mice (**D**). Data are mean + *SEM* (*n=6*). ***p*<0.028; **p*<0.048, n.s., no significance.

**
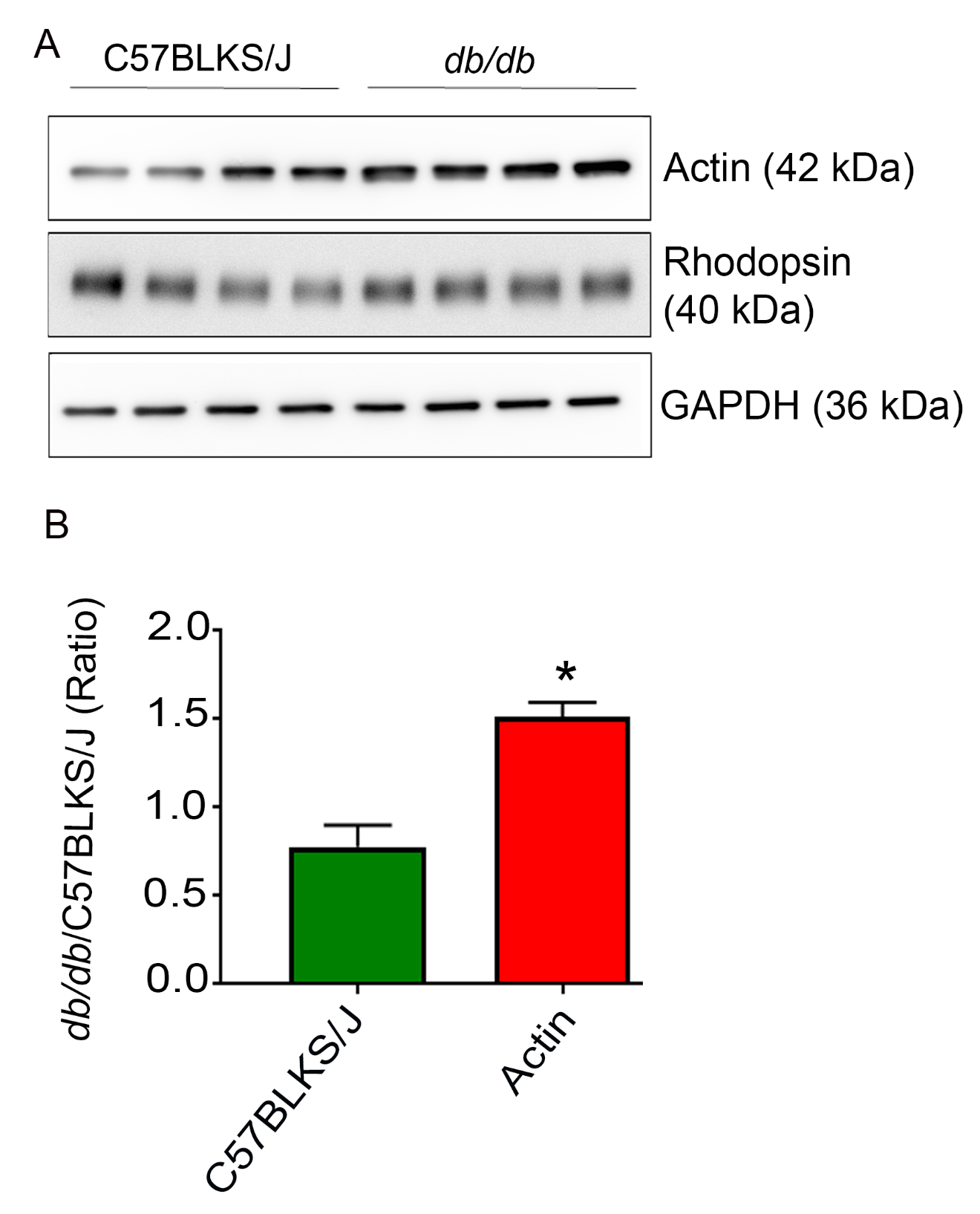
Figure S2. Increased actin levels in *db/db* mouse retina.** Retinal homogenates (10 µg protein) from C57BLKS/J and *db/db* mice were subjected to immunoblot analysis with anti-actin, anti-rhodopsin(0.5 µg), and anti-GAPDH antibodies (**A**). Densitometric analysis was carried out and we normalized the actin to GAPDH and expressed the values as a ratio (*db/db*/ C57BLKS/J) (**B**). Data are mean + *SEM* (*n=*4). **p*<0.0024. Full-length blots are presented in the Supplementary Information.

**
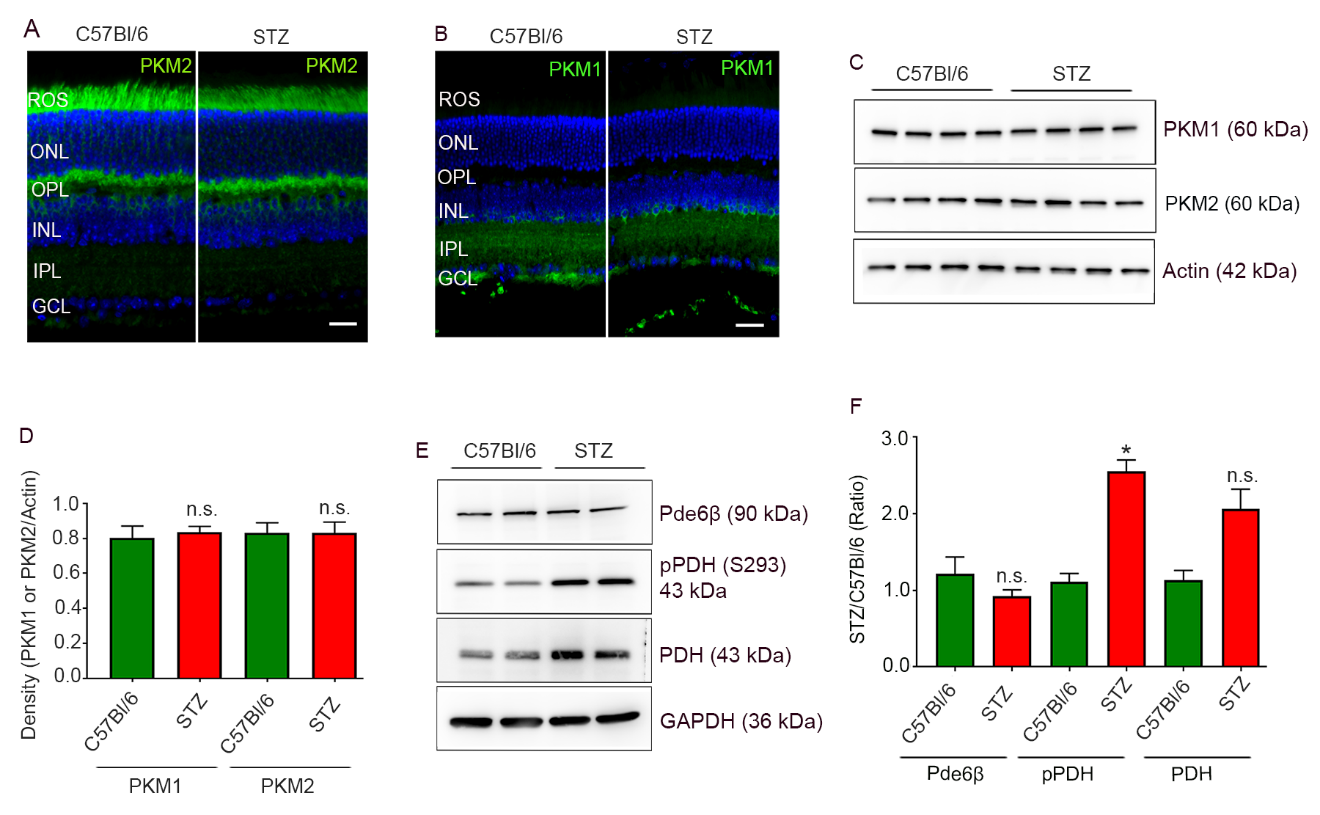
Figure S3. Expression of PKM1 and PKM2 in STZ mouse retina.** Prefer-fixed sections of C57Bl/6 and STZ-induced diabetic mouse retina were subjected to immunofluorescence with anti-PKM2 (**A**) and anti-PKM1 (**B**) antibodies. ROS, rod outer segments; RIS, rod inner segments; ONL, outer nuclear layer; OPL, outer plexiform layer; INL, inner nuclear layer; IPL, inner plexiform layer; GCL, ganglion cell layer. Scale bar = 50 μm. Retinal homogenates (10.0 µg protein) from C57Bl/6 and STZ-induced diabetic mouse retina mice were subjected to immunoblot analysis with anti-PKM1, anti-PKM2 and anti-Actin antibodies (**C**). Densitometric analysis of PKM1 and PKM2 normalized to Actin (**D**). Data are mean + *SEM* (*n=*4). Retinal homogenates (10.0 µg protein) from C57Bl/6 and STZ-induced diabetic mouse retina mice were subjected to immunoblot analysis with anti-Pde6β, anti-pPDH, anti-PDH, and anti-GAPDH antibodies (**E**). Densitometric analysis of normalized protein expression to GAPDH, and expressed the values as a ratio (STZ/control) (**F**). Data are mean + *SEM* (*n=*4). **p*<0.01; n.s., no significance. Full-length blots are presented in the Supplementary Information.

**
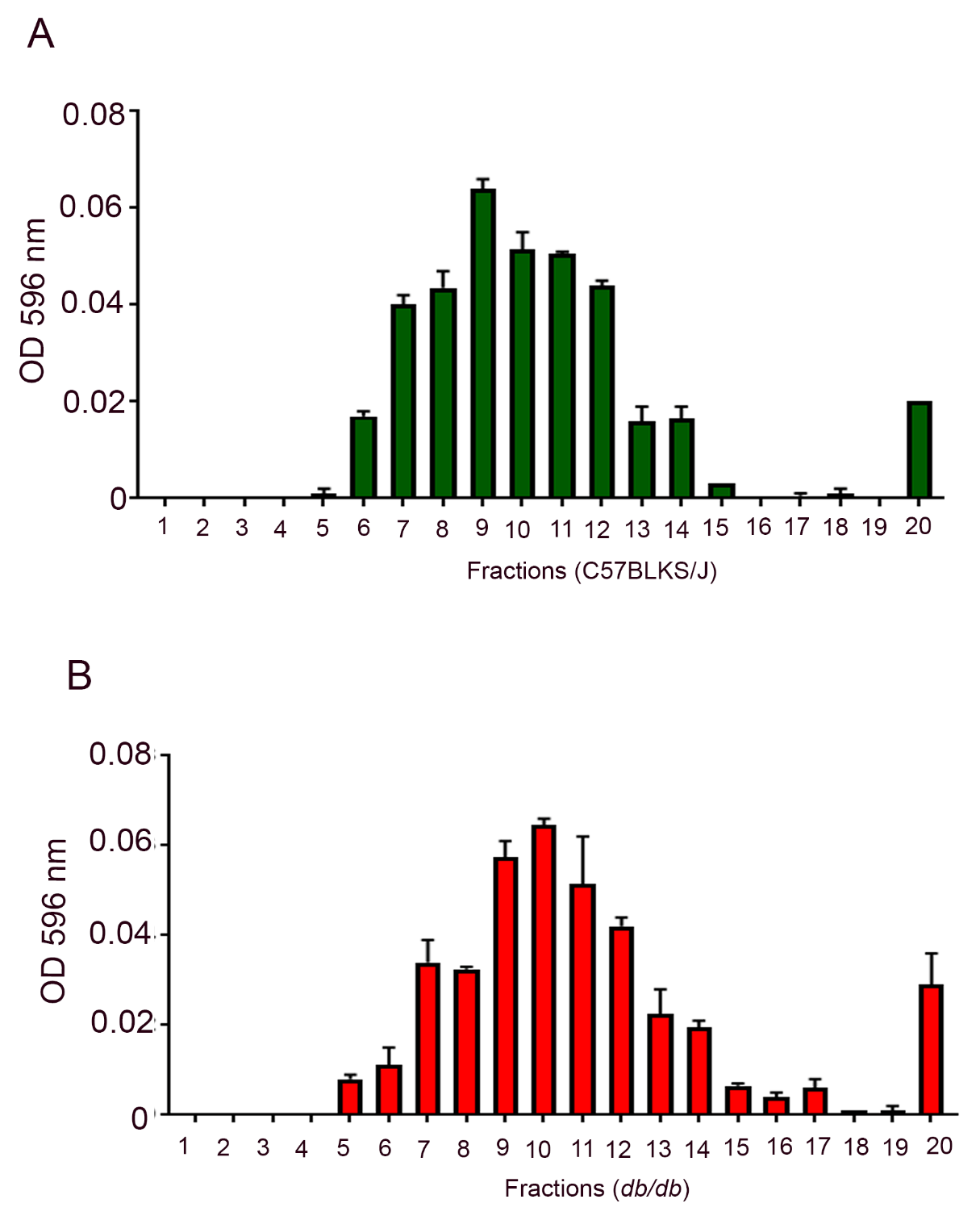
**

**Figure S4.** **Protein concentration in samples separated on glycerol density gradient centrifugation.** Protein concentration from C57BLKS/J (**A**) and *db/db* (**B**) mouse retina samples separated on glycerol gradient centrifugation were measured using BCA reagent.

**
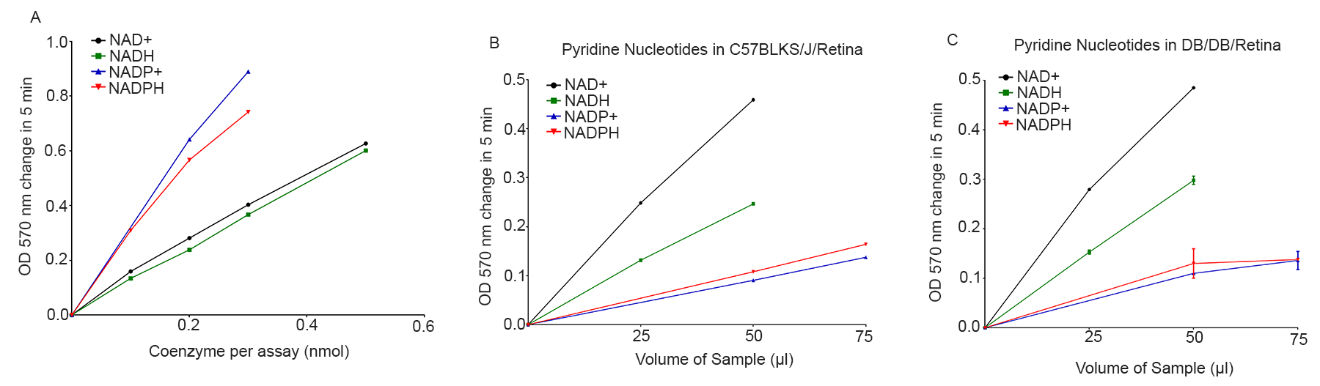
**

**Figure S5. Standard curves of NAD, NADH, NADP, and NADPH**. Standard curves for pyridine nucleotides were carried out with different concentrations of pyridine nucleotides (**A**). We also examined the linearity with different sample volumes of C57BLKS/J (**B**) and *db/db* (**C**) mouse retina.
